# MALAT1-miRNAs network regulate thymidylate synthase and affect 5FU-based chemotherapy

**DOI:** 10.1186/s10020-022-00516-2

**Published:** 2022-08-03

**Authors:** Janusz Matuszyk

**Affiliations:** Hirszfeld Institute of Immunology and Experimental Therapy, Polish Academy of Sciences, 12 R. Weigla Street, 53-114 Wroclaw, Poland

**Keywords:** Micro RNA, miRNAs, ceRNA network, 5-FU resistance, Chemoresistance, Colorectal cancer, Capecitabine, Hippo-YAP, Wnt beta-catenin

## Abstract

**Background:**

The active metabolite of 5-Fluorouracil (5FU), used in the treatment of several types of cancer, acts by inhibiting the thymidylate synthase encoded by the *TYMS* gene, which catalyzes the rate-limiting step in DNA replication. The major failure of 5FU-based cancer therapy is the development of drug resistance. High levels of *TYMS*-encoded protein in cancerous tissues are predictive of poor response to 5FU treatment. Expression of *TYMS* is regulated by various mechanisms, including involving non-coding RNAs, both miRNAs and long non-coding RNAs (lncRNAs).

**Aim:**

To delineate the miRNAs and lncRNAs network regulating the level of *TYMS*-encoded protein.

**Main body:**

Several miRNAs targeting *TYMS* mRNA have been identified in colon cancers, the levels of which can be regulated to varying degrees by lncRNAs. Due to their regulation by the MALAT1 lncRNA, these miRNAs can be divided into three groups: (1) miR-197-3p, miR-203a-3p, miR-375-3p which are downregulated by MALAT1 as confirmed experimentally and the levels of these miRNAs are actually reduced in colon and gastric cancers; (2) miR-140-3p, miR-330-3p that could potentially interact with MALAT1, but not yet supported by experimental results; (3) miR-192-5p, miR-215-5p whose seed sequences do not recognize complementary response elements within MALAT1. Considering the putative MALAT1-miRNAs interaction network, attention is drawn to the potential positive feedback loop causing increased expression of MALAT1 in colon cancer and hepatocellular carcinoma, where YAP1 acts as a transcriptional co-factor which, by binding to the TCF4 transcription factor/ β-catenin complex, may increase the activation of the *MALAT1* gene whereas the MALAT1 lncRNA can inhibit miR-375-3p which in turn targets *YAP1* mRNA.

**Conclusion:**

The network of non-coding RNAs may reduce the sensitivity of cancer cells to 5FU treatment by upregulating the level of thymidylate synthase.

## Introduction

**Fig. 1:**
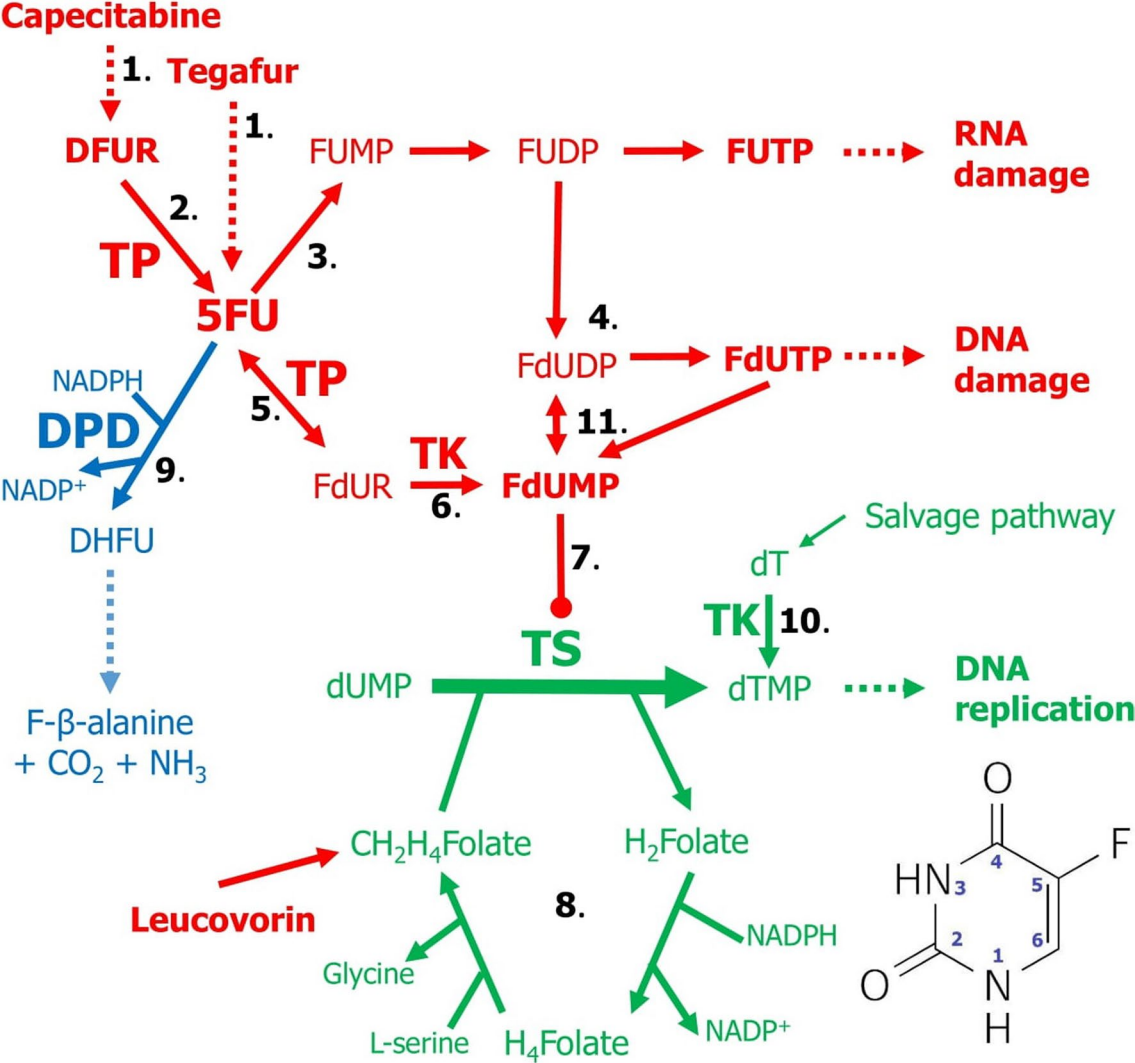
5-fluorouracil (5FU) metabolism pathways. The structure and atom numbering of 5FU is shown in the lower right corner of the figure. Antimetabolite activation pathways are marked in red. The degradation pathway for pyrimidines is marked in blue. The thymidylate synthesis is marked in green. Steps in simplified metabolic pathways: 1. Capecitabine, a 5FU prodrug, is bioactivated in the liver in a two-step process to 5’-deoxy-5-fluorouridine (doxifluridine; DFUR), while tegafur, another 5FU prodrug, is bioactivated by CYP2A6. 2. DFUR is converted to 5FU by thymidine phosphorylase (TP). 3. 5FU is sequentially converted to 5-fluorouridine monophosphate (FUMP), diphosphate (FUDP) and triphosphate (FUTP), which is an active metabolite that is mistakenly incorporated into RNA, causing RNA damage. 4. FUDP is also sequentially converted to 5-fluoro-2’-deoxyuridine diphosphate (FdUDP) and triphosphate (FdUTP), which is an active metabolite that is mistakenly incorporated into DNA, causing DNA damage. 5. TP converts 5FU to 5-fluoro-2’-deoxyuridine (FdUR). 6. Thymidine kinase (TK) converts FdUR to 5-fluoro-2’-deoxyuridine monophosphate (FdUMP). 7. FdUMP is an active metabolite that irreversibly inhibits thymidylate synthase (TS), an enzyme that catalyzes the conversion of 2’-deoxyuridine monophosphate (dUMP) to 2’-deoxythymidine monophosphate (thymidylate; dTMP). 8. Cyclically dihydrofolate (H_2_Folate) is reduced to tetrahydrofolate (H_4_Folate) which is then converted to 5,10-methylenetetrahydrofolate (CH_2_H_4_Folate). Leucovorin (folinic acid) is converted to CH_2_H_4_Folate and then stabilizes the bond formed between TS and FdUMP. 9. Dihydropyrimidine dehydrogenase (DPD) catalyzes the first step of the breakdown of 5FU (mainly in the liver) to 5,6-dihydro-5-fluorouracil (DHFU), which is further broken down into α-fluoro-β-alanine, carbon dioxide and ammonia, excreted from the body. 10. Thymidylate can be salvaged by TK from thymidine (dT) derived from dead cells. 11. FdUTP can be converted to FdUMP by dUTP pyrophosphatase and then converted back to FdUDP by UMP-CMP kinase. Based on information from Longley et al. (2003) and maps in KEGG (Kanehisa et al. 2017)

5-Fluorouracil (5FU) is a uracil analogue with a fluorine atom at the C-5 position of the pyrimidine (Fig. 1) and belongs to the class of chemotherapeutic agents known as the fluoropyrimidines. 5FU is used to treat several types of cancer. 5FU is used intravenously in palliative treatment of colorectal cancers (CRCs), breast cancers, gastric cancers, pancreatic cancers (Dean and Kane 2016a). Another fluoropyrimidine, namely capecitabine (trade name Xeloda), was rationally designed to mimic the continuous infusion of 5FU (Hoff et al. 2001) and is an oral 5FU prodrug used in the treatment of metastatic colon and breast cancers (Dean and Kane 2016b), including metastasis of HER2-positive breast cancer to the brain (Franchino et al. 2018). Tegafur, another oral prodrug of 5FU, is involved in chemotherapy for solid tumors including advanced non-small-cell lung cancer (NSCLC), CRC, gastric cancer, breast cancer, pancreatic cancer, cervical cancer (Okamoto and Fukuoka 2009). However, the therapeutic efficacy of tegafur is highly dependent on the variant of the CYP2A6 enzyme that bioactivates this prodrug (Tanner and Tyndale 2017). The anti-tumor activities of 5FU and capecitabine are exerted by the irreversible inhibition of thymidylate synthase (TS), a cytosolic enzyme encoded by the *TYMS* (Thymidylate Synthetase) gene (Fig. 1). TS catalyzes the thymidylate synthesis reaction, which is the sole source of de novo thymidylate and is the rate-limiting step in DNA replication (Longley et al. 2003). In addition, 5FU acts as an antimetabolite to replace thymine and uracil, and thus by fraudulently incorporating fluoropyrimidine metabolites into synthesized nucleic acids, it damages DNA and RNA (Blondy et al. 2020; Longley et al. 2003).

## Metabolic pathways of 5FU and capecitabine

A key enzyme in the catabolic pathway of 5FU is dihydropyrimidine dehydrogenase (DPD), encoded by the *DPYD* gene, which catalyzes the initial and rate-limiting step in the degradation of pyrimidines, including uracil, thymine, 5FU (Dean and Kane 2016a,b). DPD inactivates 5FU by catalyzing the conversion of 5FU to the non-cytotoxic metabolite 5,6-dihydro-5-fluorouracil (Fig. 1), which occurs mainly in liver cells, but also in other cells, both normal and neoplastic (Longley et al. 2003). Even more than 80% of the administered 5FU is converted in the liver to 5,6-dihydrofluorouracil (Mattison et al. 2002) and excessive DPD activity seems to be one of the mechanisms of resistance to the anti-tumor effects of 5FU (Salonga et al. 2000). On the other hand, however, special care should be taken as patients with DPD enzyme deficiency (Deac et al. 2021; Meulendijks et al. 2016) do not metabolize 5FU at a normal rate and are at risk of severe 5FU toxicity such as mucositis, diarrhea, neutropenia, and neurotoxicity (Dean and Kane 2016a,b).

In the anabolic pathway, 5FU is activated intracellularly by reactions catalyzed by two enzymes: thymidine phosphorylase (TP, encoded by the *TYMP* gene), a key enzyme that reversibly converts 5FU to 5-fluoro-2’-deoxyuridine (FdUR) and then by thymidine kinase (TK), which converts FdUR to 5-fluoro-2’-deoxyuridine monophosphate, FdUMP (Blondy et al. 2020; Longley et al. 2003), the major active metabolite of 5FU (Fig. 1) that is capable of irreversibly inhibiting TS (Langenbach et al. 1972). In contrast, capecitabine is first converted in the liver to 5’-deoxy-5-fluorouridine (doxifluridine; DFUR), which then forms 5FU upon entry into cells via a TP-catalyzed reaction (Fig. 1), which appears to be more active in cancer cells than in normal cells, possibly leading to selective accumulation of 5FU in tumors (Longley et al. 2003). Also several other enzymes convert 5FU into active antimetabolites such as 5-fluorouridine triphosphate (FUTP) and 5-fluoro-2’-deoxyuridine triphosphate (FdUTP), which due to the ability to compete with endogenous nucleosides can be misincorporated into RNA and DNA, respectively (Blondy et al. 2020; Longley et al. 2003).

## *TYMS*-encoded thymidylate synthase

TS catalyzes reductive methylation of 2’-deoxyuridine monophosphate (dUMP) to 2’-deoxythymidine monophosphate (dTMP; thymidylate) (Fig. 1), with the cofactor 5,10-methylenetetrahydrofolate (C_2_H_4_Folate) as a methyl donor (Longley et al. 2003). The TS catalyzed reaction feeds the intracellular pool of thymidylate, which is essential in DNA biosynthesis. Regarding the mechanism of TS inhibition by active fluoropyrimidine, FdUMP enters the active site in TS and forms a stable ternary complex with enzyme and cofactor, thus blocking access of dUMP to the substrate binding site in TS and consequently inhibiting thymidylate synthesis (Longley et al. 2003; Boni et al. 2010). This leads to excessive depletion of the thymidylate pool and disruption of DNA replication and repair (Longley et al. 2003). Ultimately, both thymidylate pool depletion and incorporation of fluoropyrimidine metabolites into nucleic acids lead to cell cycle arrest in S phase (Boni et al. 2010) and apoptosis in response to DNA damage.

In chemotherapy regimens, 5FU is used in combination with folinic acid, also known as leucovorin, which is converted intracellularly to 5,10-methylenetetrahydrofolate, preventing the trace release of free enzyme molecules from the enzyme-cofactor-inhibitor complex, thus enhancing the inhibitory effect of the 5FU metabolite on TS (Fig. 1). Folinic acid and 5FU in combination with oxaliplatin or irinotecan are used as the chemotherapy regimens FOLFOX and FOLFIRI, respectively, in the postoperative treatment of stage II CRC with high risk of recurrence, stage III CRC and palliative chemotherapy of metastatic stage IV CRC (Azwar et al. 2021; Iveson 2020; Taieb and Gallois 2020; Collienne and Arnold 2020; Dienstmann et al. 2015; Labianca et al. 2013; Stec et al. 2011; Grávalos et al. 2009; Van den Eynde and Hendlisz 2009). In turn, capecitabine (Xeloda) is used in combination with oxaliplatin or irinotecan as regimens XELOX (or CAPOX) and XELIRI, respectively. Evaluation of the therapeutic efficacy and toxicity of these regimens is beyond the scope of this article and is partially included in the articles cited above.

## Non-coding RNAs regulating pyrimidine metabolism and chemosensitivity to 5FU

The main failure of the fluoropyrimidine-based cancer therapy is the acquisition of drug resistance, which is a multifactorial process (Azwar et al. 2021). Among the mechanisms of resistance to 5FU, there may be changes in drug transport into and out of the cell, in particular overexpression of ATP-binding cassette transporters (Hu et al. 2016), as well as altered drug metabolism, increased level of the molecular target for an active drug, loss of cell cycle checkpoint control, increasing the threshold of apoptosis induction, the process of epithelial-mesenchymal transition (EMT) (Sun et al. 2019b), reprogramming cancer cells into cancer stem-like cells (CSCs) (Shibata et al. 2018), as well as abnormal levels of expression of non-coding RNAs (Wei et al. 2019a).

Cancer patients with low expression of all three genes *TYMP*, *DPYD* and *TYMS* had a longer survival compared to patients with high expression of any of these genes (Salonga et al. 2000). Especially high levels of *TYMS*-encoded protein in cancerous tissues are predictive of poor response to 5FU-based chemotherapy (Sakatani et al. 2019). On the other hand, higher levels of *TYMP*-encoded protein in cancer cells appear rather beneficial for capecitabine treatment as thymidine phosphorylase catalyzes the conversion of an inactive prodrug to 5FU inside the cancer cell (Terranova-Barberio et al. 2016). Interestingly, *DPYD* and *TYMS* code for enzymes that catalyze rate-limiting steps in enzyme pathways. In particular, expression of *TYMS* appears to be regulated by various mechanisms, including the involvement of non-coding RNAs. It is postulated that non-coding RNAs form a complex layer of signaling networks, important in both normal and disease processes, including cancer (Chan and Tay 2018; Irminger-Finger et al. 2014). In particular, two classes of non-coding RNAs are distinguished: short non-coding RNAs, including microRNAs (miRNAs) and long non-coding RNAs (lncRNAs) ranging from 200 to thousands of nucleotides in length (Micallef and Baron 2021; Han et al. 2015).

Mature miRNAs are single-stranded non-coding RNA molecules about 22 nucleotides in length that mediate post-transcriptional gene silencing. Currently, 1917 human hairpin precursor miRNAs and 2654 human mature miRNAs are annotated in the miRBase (22.1 release) (Kozomara et al. 2019). The synthesis of miRNA precursors and their processing in the nuclear and cytoplasmic compartments into mature miRNAs have been thoroughly described (Bartel 2018; Gebert and MacRae 2019; Saliminejad et al. 2019). Briefly, miRNA genes are transcribed into long primary miRNAs (pri-miRNAs) (Gebert and MacRae 2019). The transcripts form stem-loop structures, which are then cleaved in the nucleus by the Drosha RNase III endonuclease (complexed with two DGCR8 protein molecules, forming a so-called Microprocessor complex) into stem-loop molecules of about 70 nucleotides in length, referred to as precursor miRNAs (pre-miRNAs) (Gebert and MacRae 2019; Bartel 2018). Pre-miRNAs are transported into the cytoplasmic compartment by Exportin 5/RAN-GTP. The Dicer RNase III endonuclease then cleaves the pre-miRNA near the loop into a small miRNA:miRNA* duplex, in which the miRNA* is the opposite (passenger) strand of the miRNA (guide strand). After loading into the Argonaute protein (in the ATP-consuming process), the passenger strand of the miRNA:miRNA* duplex is discarded (Bartel 2018). A single mature miRNA is incorporated as a component of an RNA-induced silencing complex (RISC) that binds to a target RNA, primarily messenger RNA (mRNA), through complementary base pairing of a so-called seed region located between nucleotides 2–8 at the 5’-end of the miRNA molecule with a so-called microRNA response element within the target mRNA (Gebert and MacRae 2019; Bartel 2018). Capturing the targeted mRNA by the miRNA-guided Argonaute protein results in inhibition of translation or mRNA degradation mediated by RISC (Gebert and MacRae 2019).

Due to the limited number of molecules of a given type of miRNA in a cell, some lncRNAs can affect protein levels by acting as competing endogenous RNAs (ceRNAs) that bind miRNAs, preventing them from binding to targeted mRNA molecules (Chan and Tay 2018). In other words, lncRNAs can act as sponges to absorb miRNA molecules (Rashid et al. 2016; Salmena et al. 2011). Thus, among the lncRNAs with different roles in the cell (Martino et al. 2021), there may be a subset of the lncRNAs that also act as molecular decoys that sequester specific miRNAs, thereby slowing down the repression of those mRNAs targeted by these miRNAs. It can be assumed that under non-experimental conditions, due to the actual quantitative relationship in the cell between the lncRNA and the sponged miRNA, and between the miRNA and the target mRNA, a low-level miRNA in the cell will be more susceptible to lncRNA acting as a specific ceRNA (Thomson and Dinger 2016).

It was suggested that miRNAs can act as fine tuners of gene expression because targeted miRNAs are typically mildly reduced under physiological conditions (Krützfeldt 2016). A given miRNA can regulate the expression of multiple genes and it is uncertain whether its primary function is to fine-tune the entire set of different mRNAs or to suppress some major mRNAs (Lai 2015). Also, the expression of a given gene can be regulated by several different miRNAs. Accordingly, it can be expected that at least a few different miRNAs regulate the levels of the proteins encoded by *TYMS* and *DPYD*, and that altering the levels of these miRNAs may also contribute to reducing the sensitivity of cancer cells to 5FU.

## MALAT1 and other lncRNAs affecting the level of *TYMS*-encoded protein

A precursor transcript of Metastasis Associated Lung Adenocarcinoma Transcript 1 (MALAT1), also known as Nuclear-Enriched Abundant Transcript 2 (NEAT2), is almost 8.8 kb long, from which is derived an over eight kb lncRNA, which is localized in the nucleus (in nuclear specles) and involved in epigenetic modulation of gene expression and alternative splicing (Xu et al. 2022; Uthman et al. 2021). MALAT1 forms a fairly complex secondary structure with exposure of multiple miRNA-binding sites (McCown et al. 2019). Its abnormal overexpression is associated with a higher risk of metastasis in lung cancer (Karimpour et al. 2021). Interestingly, a substantially cytosolic localization of MALAT1 has been demonstrated in three colon cancer lines (HCT116, SW480, SW620) (Sun et al. 2019b), although this result will need to be confirmed in other cell lines and in CRC samples. Especially since MALAT1 is processed in the nucleus and only a short product is exported to the cytosol, while the lncRNA MALAT1 is unlikely to shuttle between the nucleus and the cytoplasmic compartment (Wilusz et al. 2008; Wilusz 2016). Importantly, however, in addition to the formation of RISC complexes in the cytoplasmic compartment, AGO2 (Argonaute protein) is also involved in various processes in the cell nucleus, including interactions with lncRNAs (Meister 2013; Li et al. 2020a) as well as some miRNAs were found in the nucleus (Gebert and MacRae 2019). An exosomal MALAT1 that can be delivered to cancer cells and sponges miRNAs has also been described (Xu et al. 2020d; Poulet et al. 2020). The indication of the cellular compartment in which MALAT1 interacts directly with various miRNAs still needs to be supported by experimental results and is under discussion (Zhou et al. 2021a; Arun et al. 2020; Sun and Ma 2019).

MALAT1 is overexpressed in many types of cancer (Goyal et al. 2021), including CRCs (Younis et al. 2022; Uthman et al. 2021; Hu et al. 2021; Zheng et al. 2020; Xiong et al. 2018; Yang et al. 2015), metastatic lung cancers (Shen et al. 2015), advanced stages of pancreatic cancers (Pang et al. 2015). In general, high levels of MALAT1 expression have been demonstrated in the vast majority of CRC specimens compared to adjacent non-tumor control colorectal tissue specimens in cancer patients (Sun et al. 2019b; Zheng et al. 2020; Luan et al. 2020; Xiong et al. 2018; Li et al. 2017a). Moreover, MALAT1 expression was significantly higher in advanced stages III–IV of tumor-node-metastasis (TNM) (Labianca et al. 2013) than in early stages I–II of CRC (Luan et al. 2020). MALAT1 has also been found to be overexpressed in human colon cancer cell lines including LoVo, SW620, SW1116, HCT116, SW480, HT29, and COLO205 cells compared to the normal human intestinal epithelial cells (Wu et al. 2018a; Tang et al. 2019a; Li et al. 2017a). However, conflicting with these observations are analyzes in another report that suggest decreased levels of MALAT1 in CRC compared to normal colon (Kwok et al. 2018).

Interestingly, the transcriptional regulator YAP1 may be significantly involved in the upregulation of *MALAT1* gene expression in cancer cells. YAP1 is an effector of the Hippo pathway and is active while the Hippo pathway is inactive (Dey et al. 2020; Wierzbicki and Rybarczyk 2015). According to the analysis of clinical data, higher levels of YAP1 were statistically significantly associated with higher stages III–IV of CRC, and statistical analysis showed a very strong positive correlation between MALAT1 and *YAP1* mRNA levels in CRC tissue specimens from patients (Sun et al. 2019b). The stability and nuclear localization of YAP1 depends on the ANKHD1 protein (ankyrin repeat and KH domain containing 1) which also acts as a YAP1 coactivator, and high levels of ANKHD1 were associated with the invasive properties of CRC cells (Almeida and Machado-Neto 2020). It turned out that nuclear YAP1 in CRC cells binds as a transcription coactivator with the TCF4/β-catenin transcription factor complex in the promoter region of the *MALAT1* gene to induce expression of this gene (Sun et al. 2019b). Interestingly, MALAT1 was shown to physically associate with the ANKHD1 protein (Yao et al. 2022), possibly via the KH domain of ANKHD1, which is involved in binding to lncRNAs (Almeida and Machado-Neto 2020). The actual location in the cellular compartment and the importance of this interaction are not fully elucidated in the cited studies of Yao et al. (2022), but it can be speculated that it may stabilize the ANKHD1 and YAP1 complex recruited by TCF4/β-catenin to the promoter region of the *MALAT1* gene and enhance gene transcription. Further, upregulated MALAT1 promotes the EMT process of CRC cells and tumor metastasis (Sun et al. 2019b; Chen and Shen 2020). In contrast, silencing MALAT1 reversed the EMT process in HT29 cells (Xiong et al. 2018), strongly arguing for a link between higher MALAT1 expression and CRC metastatic potential. The involvement of YAP1 interacting with TCF4/β-catenin in the regulation of transcription of the *MALAT1* gene was previously found in liver cancer cells (Wang et al. 2014a).

The inverse (negative) correlation of MALAT1 with a given miRNA in clinical samples may suggest a direct interaction. For example, a strong negative correlation was observed between MALAT1 and miR-200c-3p in CRCs compared to normal tissues and was associated with the response to cisplatin (Hu et al. 2021). However, clinical data should be supported by the results of reporter assays (Karreth et al. 2011; Tang et al. 2019c) using transfected cultured cells that confirm the importance of a specific site in the MALAT1 sequence for interaction with miRNA. The mentioned interaction of MALAT1 and miR-200c-3p was in fact previously confirmed in 293 T cells (Zhuo et al. 2018). It is worth mentioning here that the upregulation of MALAT1 through miR-200c-3p sponging leads to an increase in the level of the transcriptional repressor ZEB1 and contributes to the promotion of the EMT process (Pretzsch et al. 2019), and thus the migration and invasiveness of cancer cells (Zhuo et al. 2018). MALAT1 in CRCs prevents binding of miRNAs to mRNAs encoding proteins such as β-catenin, c-Myc (in the Wnt/β-catenin signaling pathway), TWIST, SLUG (promote EMT: downregulation of E-cadherin, upregulation of N-cadherin, vimentin) and many others, supported by both extensive analyzes of clinical samples and the results of experiments using established human colon cancer cell lines (Xu et al. 2022; Uthman et al. 2021). At this point, it is also worth suggesting a relationship between the level of *TYMS* expression and the EMT process in cancer cells. Namely, the results of the analysis of EMT markers in cell lines derived from tumors of various types indicate that the levels of *TYMS* mRNA are increased in cell lines with a predominance of the mesenchymal-like phenotype (Siddiqui et al. 2017). However, some caution should be exercised when comparing the results of lncRNA interaction in cell lines in vitro with respect to the interaction of the complex network of non-coding RNA in metastatic cancer cells (Witusik-Perkowska et al. 2022).

MALAT1 can also sponge several miRNAs targeting *TYMS*, as discussed later in this article. Finally, and of particular importance for further consideration, increased levels of MALAT1 in cancer cells may contribute to their resistance to 5FU chemotherapy. Namely, the level of lncRNA MALAT1 was increased in 5FU-resistant colon cancer subline HCT-116/5-FU compared to the parental HCT-116 cell line, while silencing of MALAT1 by siRNA was shown to increase the chemosensitivity of HCT-116/5-FU cells to treatment with 5FU (Tang et al. 2019a). Also in the derived HT-29FUR subline of 5FU-resistant cells, the level of MALAT1 was more than 2 times higher compared to the parent colon cancer HT-29 cell line (Aksoy et al. 2022). However, due to the involvement of MALAT1 in multiple processes, its classification as an oncogene or tumor suppressor is under discussion (Chen et al. 2020a).

Through the use of high-throughput RNA sequencing technology, it was found that several hundred lncRNAs are differentially expressed in 5FU resistant and non-resistant CRC patients. Among them, lncRNA X Inactive Specific Transcript (XIST) was found to promote TS expression through an unknown mechanism, and increased serum XIST levels were associated with lower survival rates in CRC patients receiving 5FU-based therapy (Xiao et al. 2017). The importance of XIST in CRC progression is demonstrated by increased levels of XIST in CRCs and its effect on the Wnt/β-catenin signaling pathway, which promotes elevated c-Myc levels and tumor growth (Sun et al. 2018a). Clinical data indicate that high levels of another lncRNA, namely Taurine Upregulated Gene 1 (TUG1), were also associated with recurrence of CRC in patients receiving 5FU-based chemotherapy (Wang et al. 2019a). In turn, elevated levels of lncRNA HOX Transcript Antisense RNA (HOTAIR) were also found in CRC tissues from patients and in colon cancer cell lines (Lu et al. 2018). High HOTAIR levels were associated with a poor response to 5FU treatment in CRC patients, while silencing HOTAIR in CRC cells improved their sensitivity to 5FU (Li et al. 2017b). A recent review described a number of lncRNAs acting as oncogenes in the CRCs, including HOTAIR and TUG1, which reduce chemosensitivity to 5FU (Yang et al. 2021).

## MiRNAs targeting *TYMS* mRNA

### miR-192/215-5p

*TYMS* was shown as a direct target of miR-192-5p and miR-215-5p (Boni et al. 2010; Song et al. 2010) that share the same seed region (see Table 1). Expression of miR-192/215-5p, contained in two different miRNA clusters (Vychytilova-Faltejskova and Slaby 2019), can be strongly induced in normal colon tissue by activated transcription factor p53 in response to DNA damage (Braun et al. 2008). Subsequently, miR-192/215-5p by targeting MDM2, a key p53 negative regulator (Pichiorri et al. 2010; Sun et al. 2019a), could further potentiate the action of p53, contributing to p53-dependent cell cycle arrest (Braun et al. 2008; Georges et al. 2008). It was also noted that levels of miR-192/215-5p were significantly reduced during colon carcinogenesis (Braun et al. 2008). On the other hand, an almost threefold increase in miR-215-5p levels and a dramatic decrease in the level of *TYMS*-encoded protein were detected in a small fraction of slowly proliferating colorectal cancer stem-like cells (CRC CSCs) derived from the HCT116 cell line containing wild-type p53 (Song et al. 2010). All of this together may suggest that miR-192/215-5p synchronize thymidylate synthesis with the rate of proliferation. Thus, ectopic miR-192/215-5p expression decreased the amount of *TYMS*-encoded protein in CRC cells, but this effect did not result in the expected 5FU sensitization, but paradoxically increased their resistance to 5FU treatment, presumably due to cell cycle arrest, thus reducing 5FU-sensitive fraction of cells in the S phase of the cell cycle (Boni et al. 2010). The arrest of the cell cycle was presumably a consequence of miR-215-5p targeting the DTL gene product (Denticleless protein homolog) and p53-dependent p21 upregulation as well (Fesler et al. 2015; Song et al. 2010). Interestingly, however, the increase in endogenous levels of miR-215-5p following treatment of HCT116-5FU and SW480-5FU cells (resistant to 5FU) with melatonin led to reduction in *TYMS*-encoded protein levels and could also increase the susceptibility of CRC cells to the cytotoxic effects of 5FU (Sakatani et al. 2019). Overall, miR-192/215-5p function as tumor suppressors in human cancers, including preventing the EMT process in CRC (Rokavec et al. 2019; Chen et al. 2017c), which is necessary for the initiation of the processes of migration, invasiveness and metastasis of cancer cells. On the other hand, lowering the levels of miR-192/215-5p blunts their inhibitory effects on the triglyceride synthesis pathway and genes governing extracellular matrix remodeling, which therefore promotes the progression of CRC (Zhao et al. 2019c). It should be noted, however, that miR-195/215-5p belong to several miRNAs that potentially target the largest number of genes active in CRC (Toolabi et al. 2022). Therefore, the impact of changes in these miRNA levels during therapy on tumor progression and chemoresistance may be unpredictable. Moreover, these were the conclusions of experiments carried out in well-controlled in vitro model systems, while in various clinical cases, presumably many different and uncontrolled factors affect the rate of tumor cell proliferation. However, analysis of clinical samples of post-operative CRC tissues showed that low miR-215-5p levels were significantly correlated with a high probability of 3-year recurrence, while high miR-215-5p levels could potentially predict the benefit of 5FU-based chemotherapy after surgery (Li et al. 2013). However, other reports have shown contradictory correlations between the levels of miR-215-5p and the effectiveness in the treatment of patients with CRC, which may be a result of the uncontrolled diversity of research material in many respects, including the genotype of patients and the stage of CRC (Vychytilova-Faltejskova and Slaby 2019).

**Table 1:** Confirmed miRNA response elements in the 3’-untranslated region of T*YMS* mRNA

miRNA	Sequence (5’- > 3’)^†^	Targeted sites‡	References
miR-140-3p	UACCACAGGGUAGAACCACGG	433–438	Wan et al. (2021)
miR-192-5p	CUGACCUAUGAAUUGACAGCC	97–103	Song et al. (2010)
miR-197-3p	UUCACCACCUUCUCCACCCAGC	321–327	Sun et al (2015); Wang et al (2019a)
miR-203a-3p	GUGAAAUGUUUAGGACCACUAG	291–296, 327–332	Li et al. (2015a)
miR-215-5p	AUGACCUAUGAAUUGACAGAC	97–103	Song et al. (2010)
miR-330-3p	GCAAAGCACACGGCCUGCAGAGA	192–197, 506–511	Xu et al. (2017)
miR-375-3p	UUUGUUCGUUCGGCUCGCGUGA	237–243	Xu et al. (2020b)
miR-433-3p	AUCAUGAUGGGCUCCUCGGUGU	419–426	Gotanda et al. (2013)
miR-1307-3p	ACUCGGCGUGGCGUCGGUCGUG	56–61	Chen et al. (2017a)

^†^human miRNA (hsa-miR) sequences were taken from miRBase (www.mirbase.org); seed sequences complementary to the 3’UTR of *TYMS* mRNA are underlined

^‡^3’UTR *TYMS* mRNA sites targeted by miRNA seed sequences; the numbers refer to the position after the STOP codon according to the GenBank sequence accession number NM_001071.4; the sites confirmed by the luciferase reporter assay in the cited publication are underlined

Importantly, it has recently been shown that in highly aggressive hepatocellular carcinoma (HCC), miR-215-5p targets the 3’UTR of mRNA encoding Cell Division Cycle 6 (CDC6), which is involved in the assembly of the pre-replicative complex during the G1 phase of the cell cycle (Xu et al. 2020a). In turn, it was found in breast cancer cells that miR-215-5p targets mRNA encoding RAD54 Homolog B (RAD54B), which is involved in homologous recombination repair of DNA breaks, thus inhibiting proliferation and promoting apoptosis of MCF-7 breast cancer cells (Wang et al. 2021i). It is therefore understood that lowering the levels of both miR-215-5p and miR-192-5p promotes cancer cell proliferation. Interestingly, lncRNA XIST, which is significantly elevated in hepatitis B virus-related HCC compared to adjacent liver tissues, has been found to interact with miR-192-5p and inhibit the activity of this miRNA (Wang et al. 2021h).

**Fig. 2:**
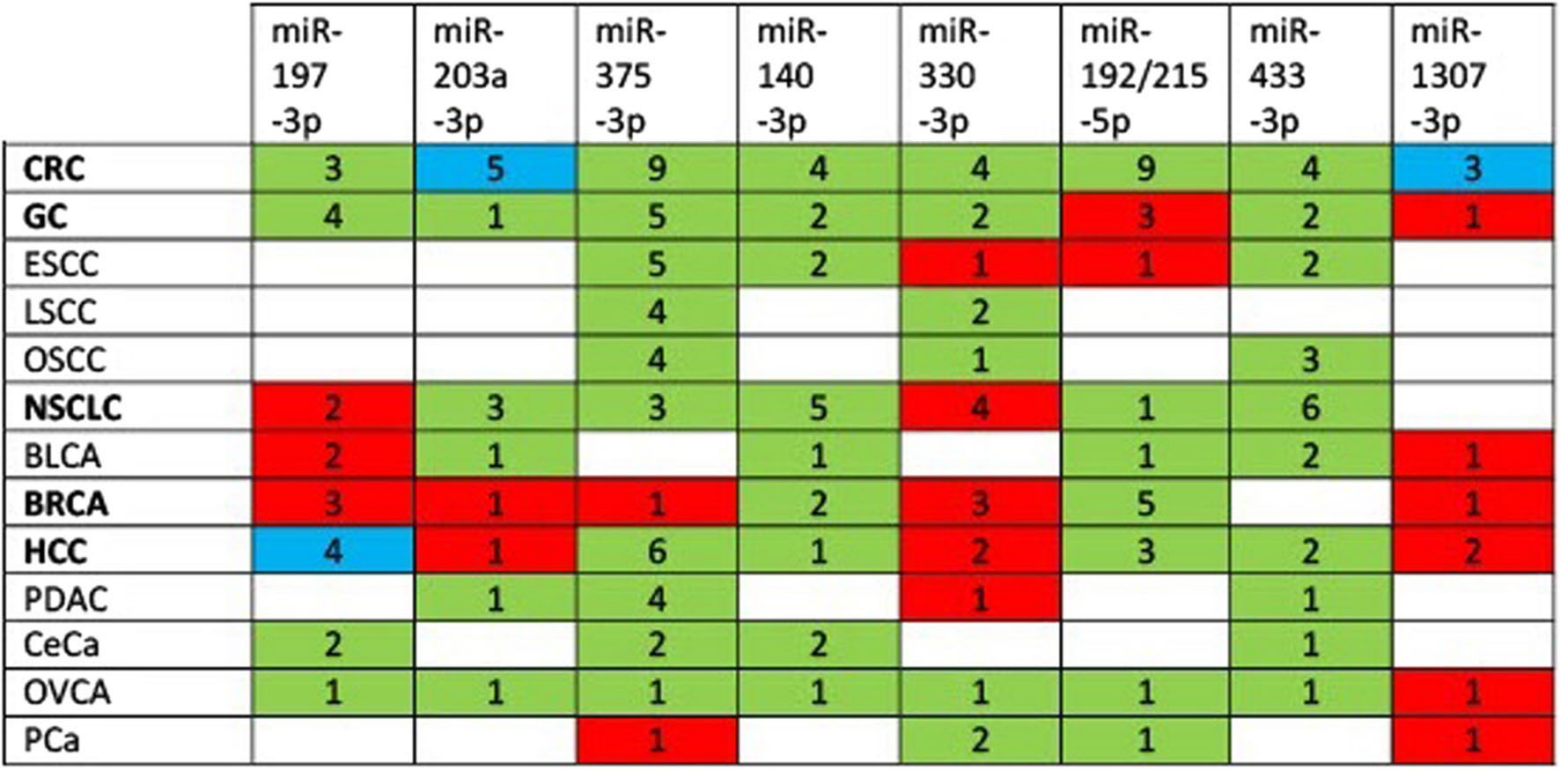
MiRNA levels in various types of cancer. Downregulated miRNAs in a given cancer type are marked green, upregulated marked red, regulated ambiguously marked blue. Cancer abbreviations as in Table 2. The numbers represent the number of the respective reports referred to in Tables 2 to 17

Overall, levels of miR-192/215-5p are down-regulated in cancers of various types (Table 2 and Fig. 2), yet up-regulated levels of miR-192/215-5p have been found in esophageal squamous cell carcinoma (ESCC) and gastric cancers (GCs) where these miRNAs also target tumor suppressors, such as mRNAs encoding the pro-apoptotic BIM protein in ESCC (Li et al. 2015c) or the important tumor suppressor RB Transcriptional Corepressor 1 (RB1) and RUNX Family Transcription Factor 1 (RUNX1) in GCs (Chen et al. 2017e; Li et al. 2016) (see also Table 3).

**Table 2:** Expression levels of miRNAs targeting i.a. *TYMS* mRNA in cancer

miRNA	Level^†^	Cancers‡	References
miR-192-5p	Down	CRC tissues	Zhao et al. (2020b)
	Down	CRC cell lines (HCT116, SW480, RKO, HT29)	Zheng et al. (2019b)
	Down	CRC tissues (TNM stage II)	Braun et al. (2008)
	Up	ESCC tissues and four cell lines	Li et al. (2015c)
	Down	NSCLC: four cell lines (i.a. A549, H1299)	Zou et al. (2019)
	Down	BLCA tissues and five cell lines (i.a. T24)	Ji et al. (2018)
	Down	BRCA tissues, MCF-7 and MDA-MB-231 cell lines	Chen et al. (2019d)
	Down	HCC tissues	Wang et al. (2021h)
	Down	HCC tissues	Lian et al. (2016)
	Down	HCC tissues	Ge et al. (2015)
	Down	PCa: PC-3 and DU145 cell lines	Sun et al. (2016)
miR-215-5p	Down	CRC tissues stages: III + IV vs I + II	Yan et al. (2020b)
	Down	CRC tissues stages: II, III, IV vs I	Vychytilova et al. (2017)
	Down	CRC tissues stages: IV vs III vs II vs I	Chen et al. (2017c)
	Down	CRC tissues: liver metastasis vs without metastasis	Chen et al. (2017c)
	Down	CRC tissues and cell lines (SW480, HCT116, LoVo, HT29)	Chen et al. (2016)
	Down	CRC tissue stages: III–IV vs I–II	Chen et al. (2016)
	Down	CRC tissues (stage II)	Braun et al. (2008)
	Down	CRC tissues	Song et al. (2010)
	Up	CRC CSCs	Song et al. (2010)
	Up	GC tissues, TNM stage III–IV vs I–II	Chen et al. (2017e)
	Up	GC tissues, stage III/IV vs I/II	Li et al. (2016)
	Up	GC tissues	Deng et al. (2014b)
	Down	BRCA tissues	Wang et al. (2021i)
	Down	BRCA tissues, stage III–IV vs I–II, three cell lines	Gao et al. (2019)
	Down	BRCA tissues, cell lines (i.a. MDA-MB-231)	Yao et al. (2017)
	Down	BRCA tissues	Zhou et al. (2014a)
	Down	OVCA tissues and three cell lines	Ge et al. (2016)

^†^”UP” or “down” in the level of a given miRNA: in cancerous tissues as compared to adjacent non-cancerous tissues (or to tissues of healthy individuals in cases of cervical and prostate cancers); one stage of cancer compared to another stage; cancer cell line compared to a normal epithelial cell line

^‡^Cancer tissues collected from patients and cancer cell lines of various types: *BLCA* bladder cancer, *BRCA* breast cancer (various molecular subtypes including triple-negative breast cancer TNBC), *CeCa* cervical cancer, *CRC* colorectal cancer, *ESCC* esophageal squamous cell carcinoma, *GC* gastric cancer, *HCC* hepatocellular carcinoma, *LSCC* laryngeal squamous cell carcinoma, *NSCLC* non-small cell lung cancer (including *LUAD* lung adenocarcinoma *SqCLC* squamous cell lung carcinoma), *OVCA* ovarian cancer, *OSCC* oral squamous cell carcinoma, *PCa* prostate cancer, *PDAC* pancreatic ductal adenocarcinoma

)

**Table 3:** Major genes targeted by miR-192-5p and miR-215-5p in various types of cancer

Cancers	Targeted gene products^†^	References
Cancers with down-regulation of miR-192/215-5p		
	*Acting as oncogenes or tumor promoters*	
CRC	TYMS, *EIF5A2*, *EREG*, *HOXB9*	Boni et al. (2010); Song et al. (2010); Zhao et al. (2020b); Chen et al. (2016)
	*RAB2A*, *YY1*, *ZEB2*	Vychytilova et al. (2017); Zheng et al. (2019b); Chen et al. (2017c)
BRCA	*AKT1*, *RAD54B*, *SOX9*	Yao et al. (2017); Wang et al. (2021i); Gao et al. (2019)
NSCLC	*TRIM44*	Zou et al. (2019)
BLCA	*YY1*	Ji et al. (2018)
HCC	*CDC6*, *SLC39A6*, *TRIM25*	Xu et al. (2020a); Lian et al. (2016); Wang et al. (2021h)
PCa	*NOB1*	Sun et al. (2016)
Cancers with up-regulation of miR-192/215-5p		
	*Acting as tumor suppressors*	
GC	*RB1*, *RUNX1*	Chen et al. (2017e); Deng et al. (2014b); Li et al. (2016
ESCC	*BCL2L11*	Li et al. (2015c)

^†^mRNAs encoding the following proteins: *AKT1* encodes AKT Serine/Threonine Kinase 1; *BCL2L11* codes for pro-apoptotic BH3-only protein BIM; *CDC6* is an oncogene encoding Cell Division Cycle 6 Homolog; *EIF5A2* is an oncogene encoding Eukaryotic Translation Initiation Factor 5A2; *EREG* codes for Epiregulin, ligand of epidermal growth factor receptor (Cheng et al. (2021); *HOXB9* encodes transcription factor Homeobox B9 (Contarelli et al. 2020); *NOB1* is an oncogene encoding Nin One Binding Protein; *RAB2A* is an oncogene encoding a member of the RAS family; *RAD54B* is an oncogene encoding helicase RAD54 Homolog B involved in homologous recombination and DNA repair (Zhang et al. 2019c); *RB1* is a tumor suppressor gene encoding RB Transcriptional Corepressor 1, a key regulator of the G1/S transition in the cell cycle; *RUNX1* is tumor suppressor gene coding for RUNX Family Transcription Factor 1; *SLC39A6* is an oncogene that encodes a zinc-influx transporter; *SOX9* is an oncogene encoding SRY-Box Transcription Factor 9; *TRIM25* encodes E3 ubiquitin ligase involved in p53 inactivation (Zhang et al. 2015); *TRIM44* is an oncogene encoding a protein involved in the ubiquitination and degradation of target proteins; *YY1* is an oncogene encoding Yin Yang 1 transcriptional factor; *ZEB2* encodes Zinc Finger E-Box Binding Homeobox 2, a transcriptional corepressor promoting the EMT process in cancer cells

Interestingly, in addition to miR-215-5p targeting *TYMS* expression, also miR-215-3p derived from the opposite arm of the same primary hairpin microRNA has gene silencing activity that influences 5FU cytotoxic activity since ectopic overexpression of miR-215-3p increased the sensitivity of CRC cells to 5FU, and although the exact mechanism has not been discovered, it has been shown to be at least in part related to targeting the CXCR1 chemokine receptor, which is an interleukin-8 receptor (Li et al. 2018a).

As show in Table 3, miR-192/215-5p down-regulation in CRCs not only promotes an increase in TS, but also an increase in ZEB2, which promotes the EMT process (Pretzsch et al. 2019), and an increase in eIF5A2, which further potentiates the EMT process (Bao et al. 2015). Down-regulation of miR-192/215-5p are generally found in cancers of various types, and in breast cancer it results in an increase in the levels of AKT protein kinase and DNA repair and recombination protein RAD54B.

Finally, it is worth mentioning that p53, which raises the level of miR-192/215-5p, may also act as a transcription repressor of the *MALAT1* gene, at least in hematopoietic cells (Ma et al. 2015). As counteraction, MALAT1 may indirectly deacetylate p53 and inhibit p53 transcriptional activity as found in HepG2 cells (Chen et al. 2017h).

Reports also indicate several other miRNAs, which in various types of cancer may regulate the level of protein encoded by *TYMS* and thus affect the sensitivity of cancer cells to 5FU chemotherapy, including miRNA such as miR-140-3p (Wan et al. 2021), miR-197-3p (Sun et al. 2015), miR-203a-3p (Li et al. 2015a), miR-218-5p (Li et al. 2015b), miR-330-3p (Xu et al. 2017), miR-375-3p (Xu et al. 2020b), miR-433-3p (Gotanda et al. 2013).

### MiR-140-3p

MiR-140-3p has been reported to target the 3’UTR of *TYMS* mRNA in lung adenocarcinoma cells (Wan et al. 2021). Interestingly, miR-140-5p levels were also reported to be decreased in CRC specimens taken from patients, but increased in slowly proliferating and chemoresistant CRC CSCs, and furthermore, CRC cells transfected with miR-140 precursor were resistant to 5FU (Song et al. 2009). However, it is still worth awaiting stronger experimental support for a direct link between levels of miR-140-3p and *TYMS*-encoded protein, and the chemosensitivity of CRC cells to 5FU. It is worth noting that miR-140-3p also has the potential to target mRNA encoding Phosphatase and TENsin homolog (PTEN) (Yin et al. 2020), an important tumor suppressor that antagonizes the effects of PI3K, thereby activating the PI3K/AKT signaling pathway, although it appears that this putative miR-140-3p function in cancer cells is not likely to be significant. As shown in Table 4, miR-140-3p is generally down-regulated in various types of cancer, causing, in addition to the expected increase in TS, also an increase in the anti-apoptotic protein BCL-2 in CRC and GC cells and activation of the WNT/β-catenin pathway in CRC (see Table 5).

**Table 4:** Expression levels of miRNAs targeting i.a. *TYMS* mRNA in cancer

miRNA	Level†	Cancers^‡^	References
miR-140-3p	Down	CRC tissues	Chen et al. (2020c)
	Down	CRC tissues, SW480 and HCT116 cell lines	Jiang et al. (2019a)
	Down	Primary tumor tissues of CRC patients	Piepoli et al. (2012)
	Down	Liver metastatic vs none-metastatic CRC	Liu et al. (2021)
	Down	GC tissues and cell lines	Wang et al. (2021a)
	Down	GC tissues and five cell lines	Chen et al. (2021)
	Down	ESCC tissues and five cell lines	Wang et al. (2021d)
	Down	ESCC tissues and five cell lines	Chen et al. (2020d)
	Down	NSCLC (LUAD) tissues and five cell lines (i.a. A549)	Wang et al. (2021f)
	Down	NSCLC (LUAD): four cell lines (i.a. A549, H1299)	Wu et al. (2020)
	Down	NSCLC tissues and three cell lines (i.a. A549, H1299)	Hu et al. (2020)
	Down	NSCLC (SqCLC) tissues, stage III vs II vs I, four cell lines	Huang et al. (2019a)
	Down	NSCLC tissues	Dong et al. (2016)
	Down	Lung cancer tissues and six cell lines (i.a. A549)	Kong et al. (2015)
	Down	BLCA tissues and four cell lines (i.a. T24)	Yuan et al. (2021)
	Down	BRCA tissues stage I-IV	Dou et al. (2021)
	Down	BRCA tissues, MCF-7 (ER^+^) and MDA-MB-453 (HER2^+^)	Zhou et al. (2019)
	Down	HCC tissues	Gao et al. (2021b)
	Down	CeCa tissues	Wang et al. (2022a)
	Down	CeCa tissues	Wang et al. (2021g)
	Down	OVCA tissues	Qiao et al. (2021)

^†^,^‡^For description see footnote to Table 2

**Table 5:** Major genes targeted by miR-140-3p in various types of cancer

Cancers	Targeted gene products^†^	References
Cancers with down-regulation of miR-140-3p		
	*Acting as oncogenes or tumor promoters*	
CRC	*BCL2*, *BCL9*	Liu et al. (2021)
GC	*BCL2*	Chen et al. (2021)
ESCC	*E2F3*, *NRIP1*	Wang et al. (2021d); Chen et al. (2020d)
LUAD	TYMS	Wan et al. (2021)
Lung cancer	*ATP6AP2*, *BRD9*, *JAK1*	Kong et al. (2015); Huang et al. (2019a); Hu et al. (2020)
BLCA	*ANXA8*	Yuan et al. (2021)
HCC	*GRN*, *VEGFA*	Gao et al. (2021b); Hou et al. (2020)
OVCA	*AGTR1*	Qiao et al. (2021)
	*Acting both as oncogenes and tumor suppressors, or unclassified*	
LUAD	*E2F7*	Wang et al. (2021f)
BRCA	*TRIM28*	Zhou et al. (2019)
CeCa	*ELOA* (*TCEB3*), *PDZK1*	Wang et al. (2021g); Wang et al. (2022a)

^†^mRNAs encoding the following proteins: *AGTR1* encodes the Angiotensin II Receptor Type I and is recognized as an oncogene in various cancers; *ANXA8* encodes Annexin A8 that is highly expressed in some cancers; *ATP6AP2* is a proto-oncogene coding for ATPase H + Transporting Accessory Protein 2 (Renin/Prorenin Receptor); *BCL2* codes for anti-apoptotic protein; *BCL9* codes for the coactivator involved in β-catenin mediated transcription; BRD9 encodes Bromodomain-Containing Protein 9 involved in chromatin remodeling complexes; *E2F3* encodes E2F Transcription Factor 3; *E2F7* is a tumor suppressor gene that encodes atypical E2F Transcription Factor 7; ELOA encodes Elongin A, also known as RNA polymerase II Transcription Elongation Factor B Subunit 3; *GRN* encodes Granulin Precursor that is presumably involved in tumorigenesis; *JAK1* encodes Janus Kinase 1 phosphorylating a tyrosine residue; *NRIP1* encodes Nuclear Receptor Interacting Protein 1 which is elevated in tumors; *PDZK1* is a tumor suppressor gene encoding PDZ Domain Containing 1, scaffolding protein; *TRIM28* encodes a protein that may have both oncogenic and tumor suppressor effects; *VEGFA* encodes Vascular Endothelial Growth Factor A that promotes angiogenesis

Potentially both miR-140-5p and miR-140-3p could be sponged by lncRNA MALAT1, although this has not been shown in CRC, while the direct interaction of miR-140-5p with MALAT1 has been shown experimentally in cancers of various types such, as prostate cancer (Hao et al. 2020), hepatocellular carcinoma (Fan et al. 2020; Hou et al. 2020), osteosarcoma (Sun and Qin 2018), tongue squamous cell carcinoma (Zhu et al. 2019). MiR-140-3p levels can also be regulated by lncRNA TUG1 and this has already been demonstrated in bladder cancer (Yuan et al. 2021). It was also confirmed that TUG1 sponges miR-140-5p in osteosarcoma cells (Zhao et al. 2019a).

### MiR-197-3p

One report showed that miR-197-3p directly targets the 3’UTR of *TYMS* mRNA, which led to a reduction in TS protein level, while increasing the sensitivity of CRC cells to the cytotoxic effects of 5FU (Sun et al. 2015). On the other hand, lncRNA TUG1 can act as a ceRNA that sponges miR-197-3p and thereby increases the level of *TYMS*-encoded protein, mediating the acquisition of 5FU resistance by CRC cells (Wang et al. 2019a). HOTAIR, which is elevated in CRC tissues, has also been shown to sponge miR-197-3p (Lu et al. 2018). Importantly, miR-197-3p has been found to be sponged also by MALAT1 in NSCLC, contributing to the process of EMT and cancer cell resistance to treatment with cisplatin, adriamycin, gefitinib and paclitaxel (Yang et al. 2019). The direct interaction of miR-197-3p with the miRNA response elements in MALAT1 was confirmed by the results of reporter assays (Yang et al. 2019). In this context, it would be particularly valuable to determine whether the increase in the level of MALAT1 in CRCs contributes to the reduction of their chemosensitivity to 5FU, e.g. by sponging miR-197-3p. According to old measurements, the level of miR-197-3p was decreased in the HCT116 colorectal cancer cell line after treatment with 5FU (Zhou et al. 2010). It is worth noting at this point that SGC7901/5-FU gastric cancer cells partially resistant to 5FU also had decreased level of miR-197-3p compared to parental SGC7901 line, while transfection of the miR-197 mimic into SGC7901/5-FU cells restored sensitivity to the growth inhibitory effects of 5FU (Xiong et al. 2015). Importantly, miR-197-3p, whose levels are lower in CRCs (Lu et al. 2018), in turn in NSCLC, acts as an oncomiR targeting mRNAs encoding pro-apoptotic BH3-only proteins NOXA and BMF (Fiori et al. 2014).

As shown in Tables 6 and 7, miR-197-3p is down-regulated in CRC and GC, where it targets *TYMS* and *MTDH*, which encodes a protein also involved in *TYMS* induction and *PTEN* repression, while in NSCLC and BRCA miR-197-3p is up-regulated because it also targets suppressor genes that are important in these cancers. Reports on the level of miR-197-3p in HCC seem contradictory, but it is worth paying attention to the up-regulation of the level of miR-197-3p in metastatic HCC, which results in a decrease in the level of negative regulators of the WNT/β-catenin pathway (AXIN2, NKD1, DKK2) in HCC clinical tissues (Hu et al. 2018a).

**Table 6:** Expression levels of miRNAs targeting i.a. T*YMS* mRNA in cancer

miRNA	Level^†^	Cancers^‡^	References
miR-197-3p	Down	HCT8Fu (5FU resistant) vs HCT8 (parental sensitive)	Wang et al. (2019a)
	Down	CRC tissues, HCT116, LoVo, HT29, SW480 cell lines	Lu et al. (2018)
	Down	after treatment with 5FU in HCT116 cell line	Zhou et al. (2010)
	Down	GC tissues and two cell lines	Han and Liu (2021)
	Down	GC tissues	Niu et al. (2020)
	Down	GC tissues	Chen et al. (2019c)
	Down	GC tissues and four cell lines	Liao et al. (2018)
	Up	NSCLC tissues and four cell lines (i.a. A549, H460, H1299)	Yang et al. (2019)
	Up	NSCLC (LUAD) tissues	Chen and Yang (2018)
	Up	BLCA tissues and four cell lines (i.a. T24)	Jiang et al. (2019b)
	Up	BLCA tissues and three cell lines (i.a. T24)	Wang et al. (2016a)
	Up	BRCA tissues and MCF-7, T47D cell lines (ER^+^ luminal A)	Li et al. (2021d)
	Up	BRCA 11 cell lines, TNBC vs luminal	Ye et al. (2019)
	Up	BRCA (TNBC) tissues	Tang et al. (2018)
	Down	HCC tissues	Bi et al. (2021)
	Down	HCC tissues and three cell lines	Ni et al. (2019)
	Up	HCC tissues with metastasis vs non-metastasis	Hu et al. (2018a)
	Up	HCC cell lines	Dai et al. (2014)
	Down	CeCa tissues	Gu et al. (2021)
	Down	CeCa tissues and four cell lines	Hu et al. (2018b)
	Down	OVCA tissues and four cell lines	Xie et al. (2020)

^†^,^‡^For description see footnote to Table 2

**Table 7:** Major genes targeted by miR-197-3p in various types of cancer

Cancers	Targeted gene products^†^	References
Cancers with down-regulation of miR-197-3p		
	*Acting as oncogenes or tumor promoters*	
CRC	TYMS	Sun et al. (2015); Wang et al. (2019a)
GC	*PRKCB*	Chen et al. (2019c)
GC	*CXCR6*, *MAPK1*, *MTDH*	Han and Liu (2021); Xiong et al. (2015); Liao et al. (2018)
HCC	*AGR2*	Bi et al. (2021)
CeCa	*E2F6*, *FOXM1*	Gu et al. (2021); Hu et al. (2018b)
	*Acting both as oncogenes and tumor suppressors, or unclassified*	
HCC	*ZIK1*	Ni et al. (2019)
OVCA	*ABCA7*	Xie et al. (2020)
Cancers with up-regulation of miR-197-3p		
	*Acting as tumor suppressors*	
NSCLC	*BMF*, *PMAIP1* (NOXA)	Fiori et al. (2014)
LUAD	*CYLD*	Chen and Yang (2018)
BRCA	*FBXW7*, *HIPK3*, *NLK*	Ye et al. (2019); Li et al. (2021d); Tang et al. (2018)
HCC	*AXIN2*, *NKD1*, *DKK2*, *CD82*	Hu et al. (2018a); Dai et al. (2014)
	*Acting both as oncogenes and tumor suppressors, or unclassified*	
NSCLC	*CTNND1*	Yang et al. (2019)

^†^mRNAs encoding the following proteins: *ABCA7* encodes ATP Binding Cassette Subfamily A Member 7 (Muriithi et al. 2020); *AGR2* is an oncogene that encodes a protein disulphide isomerase; *AXIN2*, *NKD1* and *DKK2* function as tumor suppressors and regulators of the WNT/β-catenin pathway; *BMF* encodes pro-apoptotic BH3-only protein; *CD82* codes for metastasis suppressor; *CTNND1* encodes Catenin Delta 1 which interacts with cadherins and acts in adhesion between cells; *CXCR6* encodes C-X-C Motif Chemokine Receptor 6; *CYLD* is a tumor suppressor gene that codes for CYLD Lysine 63 Deubiquitinase; *E2F6* encodes E2F Transcription Factor 6; *FBXW7* is a tumor suppressor gene encoding F-Box and WD Repeat Domain Containing 7 which targets cyclin E and c-Myc for ubiquitin-mediated degradation; *FOXM1* functions as an oncogene encoding Forkhead Box M1 protein; *HIPK3* codes for Homeodomain Interacting Protein Kinase 3, serine/threonine protein kinase; *MAPK1* encodes Mitogen-Activated Protein Kinase 1 (p42-MAPK/ERK2); *MTDH* (Metadherin) acts as an oncogene and is involved in induction of *TYMS* (Yoo et al. 2009); *NLK* codes for Nemo-Like Kinase, serine/threonine protein kinase and acts as tumor suppressor in breast cancer; *PMAIP1* codes for NOXA, which is a p53-induced pro-apoptotic BH3-only protein; *PRKCB* codes for Protein Kinase Cβ; *ZIK1* encodes the Zinc Finger Protein Interacting With K Protein 1, which probably acts as a transcriptional repressor

### MiR-203a-3p

Levels of miR-203a-3p were also found to be decreased in 5FU-resistant CRC cells (see Table 8). Mir-203a-3p can target several sites in the 3’UTR of *TYMS* mRNA (Table 1) and it was found that silencing of miR-203a-3p by an antisense oligonucleotide increased the level of the *TYMS*-encoded protein and decreased the sensitivity of CRC cells to the cytotoxic effects of 5FU, and conversely, as a result of miR-203a mimic transfection, a decreased level of TS protein and an increased sensitivity to 5FU were observed (Li et al. 2015a). More importantly, miR-203a-3p increased the anti-tumor activity of 5FU also after injection of the miR-203 precursor along with 5FU into NOD/SCID mice with CRC (Li et al. 2015a). Importantly, however, miR-203a-3p can also target mRNA encoding PTEN, thereby activating the PI3K/AKT signaling pathway, and its involvement in stimulating cell proliferation and inhibiting apoptosis has been demonstrated in hepatocytes (Zhang et al. 2020).

**Table 8:** Expression levels of miRNAs targeting i.a. T*YMS* mRNA in cancer

miRNA	Level^†^	Cancers^‡^	References
miR-203a-3p	Down	CRC tissues, SW480, HT29, SW620, HCT15 cell lines	Qian et al. (2019)
	Up	CRC tissues, HCT116, HT29, LoVo, SW1116 cell lines	Chen et al. (2018)
	Down	CRC tissues	Xiao et al. (2018)
	Down	LoVo/5-FU (5FU resistant) vs LoVo (parental sensitive)	Li et al. (2015a)
	Up	in serum of CRC patients	Huang et al. (2020a)
	Down	GC tissues and two cell lines	Wang et al. (2018a)
	Down	NSCLC tissues	Liang et al. (2020)
	Down	NSCLC tissues	Yang et al. (2020)
	Down	NSCLC (LUAD) tissues, TNM stage III–IV vs I–II	Wang et al. (2020a)
	Down	BLCA tissues and four cell lines (i.a. T24)	Na et al. (2019)
	Up	BRCA tissues (mainly luminal A/B)	Gomes et al. (2016)
	Up	HCC tissues	Huo et al. (2017)
	Down	PDAC four cell lines (i.a. PANC-1, SW 1990)	An and Zheng (2020)
	Down	OVCA tissues	Liu et al. (2019a)

^†^,^‡^For description see footnote to Table 2

Moreover, it has been shown in ovarian cancer and earlier in glioblastomas that miR-203a-3p can target mRNA encoding ataxia-telangiectasia (ATM) (Liu et al. 2019a; Yang et al. 2017), a serine-threonine kinase activated by a double-strand DNA break and involved in the checkpoint of DNA damage response processes. This has potential implications for therapy as inhibition of ATM has been shown to sensitize gliomas to chemotherapy (Yang et al. 2017).

In addition to the brief summary in Table 9, it is worth noting that miR-203a-3p targets *TYMS* mRNA and a number of oncogenes in various types of cancer, such as *BIRC5* encoding the anti-apoptotic Survivin and *SNAI2*/*ZEB2* encoding transcriptional repressors that promote EMT (Pretzsch et al. 2019). On the other hand, miR-203a-3p can also target tumor suppressors and is up-regulated in HCC (Tables 8, 9).

**Table 9:** Major genes targeted by miR-203a-3p in various types of cancer

Cancers	Targeted gene products^†^	References
Cancers with down-regulation of miR-203a-3p		
	*Acting as oncogenes or tumor promoters*	
CRC	TYMS	Li et al. (2015a)
Glioblastoma	TYMS	Chen et al. (2017b)
NSCLC	*AVL9*, *BIRC5*, *DLX5*, *E2F1*, *ZEB2*	Liang et al. (2020); Yang et al. (2020); Wang et al. (2020a)
BLCA	*SIX4*	Na et al. (2019)
PDAC	*SNAI2*	An and Zheng (2020
	*Acting both as oncogenes and tumor suppressors, or unclassified*	
CRC	*THBS2*	Qian et al. (2019)
GC	*IGF1R*	Wang et al. (2018a)
OVCA	*ATM*	Liu et al. (2019a)
Cancers with up-regulation of miR-203a-3p		
	*Acting as tumor suppressors*	
HCC	*IL24*, *PTEN*	Huo et al. (2017); Zhang et al. (2020)
	*Acting both as oncogenes and tumor suppressors, or unclassified*	
CRC	*PDE4D*	Chen et al. (2018)

^†^mRNAs encoding the following proteins: *ATM* codes for ATM Serine/Threonine Kinase (Ataxia Telangiectasia Mutated), although it is considered as a tumor suppressor, ATM signaling can be involved in chemoresistance of cancer cells (Cremona and Behrens 2014) as well as promote the EMT process (Liu et al. 2019a); *AVL9* codes for cell migration associated protein and is an oncogene in NSCLC; *BIRC5* can be regarded as an oncogene that codes for apoptosis inhibitor Survivin; *DLX5* codes for transcriptional activator and acts as oncogene (Tan and Testa 2021); *E2F1* encodes E2F Transcription Factor 1; *IGF1R* encodes the Insulin-like Growth Factor 1 Receptor (Werner et al. 2016); *IL24* codes for interleukin 24, which can induce apoptosis in a variety of cancer cells, thereby acting as a tumor suppressor; *PDE4D* encodes cAMP-specific Phosphodiesterase 4D; *PTEN* is a tumor suppressor gene encoding Phosphatase and Tensin Homolog; *SIX4* codes for transcriptional regulator and acts as oncogene; *THBS2* codes for Thrombospondin 2; *SNAI2* codes for SLUG which represses the gene encoding E-cadherin and thus promotes the EMT process of cancer cells

Interestingly, lncRNA MALAT1 was shown to downregulate miR-203a-3p levels in glioblastoma multiforme cells, thereby promoting *TYM*S expression (Chen et al. 2017b). The direct interaction of MALAT1 and miRNA was indicated by the significant upregulation of miR-203a-3p in si-MALAT1 transfected human glioblastoma cells resistant to temozolomide (Chen et al. 2017b). Other studies confirmed that MALAT1 sponges miR-203a-3p in renal cell carcinoma (Zhang et al. 2019a). Luciferase reporter assays confirmed a targeted relationship between MALAT1 and miR-203a-3p, and expression level analyzes showed significant upregulation of miR-203a-3p in si-MALAT1 transfected renal cell carcinoma lines (Zhang et al. 2019a). The direct interaction of miR-203a-3p with two sites in MALAT1 was also demonstrated in luciferase reporter assays in human retinal microvascular endothelial cells (Yu et al. 2020). RT-PCR analysis of CRC samples from 85 patients also showed an inverse correlation between MALAT1 and miR-203a-3p (Wu et al. 2018b). However, the question of localization in the cellular compartment where the demonstrated direct interaction of MALAT1-miRNA takes place still needs to be answered.

### MiR-218-5p

MiR-218-5p was found to be downregulated in primary CRC tissues and its expression in CRC cell lines was significantly decreased after treatment with 5FU, while ectopic overexpression of miR-218-5p directly suppressed *BIRC5*-encoded Survivin as well as indirectly decreased TS levels through an unknown molecular mechanism (Li et al. 2015b). MiR-218-5p can be derived from two stem-loop sequences, mir-218-1 and mir-218-2, which are transcribed from two loci located on chromosomes 4p15.31 and 5q35.1, respectively (Guan et al. 2013), and its level may be partially regulated by lncRNA HOTAIR which can recruit EZH2 (enhancer of zeste 2 polycomb repressive complex 2 subunit) to the promoter of the *SLIT3* gene and repress its transcription along with the mir-218-2 contained therein (Li et al. 2017b). Importantly, increased levels of MALAT1 in CRCs have been shown to directly inhibit the activity of miR-218-5p as well as cause EZH2-dependent repression of the *CDH1* gene encoding E-cadherin (Li et al. 2017a). Thus, by repressing the *CDH1* gene and sponging the miR-218-5p, MALAT1 can promote the EMT process and cancer cell metastasis, lead to an increase in Survivin levels and cancer cell resistance to 5FU and oxaliplatin treatment.

### MiR-330-3p

Other studies found that miR-330-3p levels were reduced in CRC tissues (see Table 10) compared to adjacent normal tissues in patients, suggesting that miR-330-3p may act as a tumor suppressor (Xu et al. 2017). Importantly, miR-330-3p levels were also decreased in 5FU-resistant CRC tissues compared to 5FU-sensitive CRC tissues after surgery (Gao et al. 2021a). Moreover, it was shown that ectopic expression of miR-330 mimics directly decreased the level of *TYMS*-encoded protein and increased the sensitivity of CRC cells to the cytotoxic effect of 5FU (Xu et al. 2017). Also, the second strand of the mature miRNA, namely miR-330-5p, has the potential to regulate *TYMS* expression because the miRNA response element is located in the mRNA coding region, but the direct interaction of miR-330-5p with *TYMS* mRNA still needs to be supported by experimental results. MiR-330-5p levels have been found to be reduced in CRC tissues and two colon cancer cell lines (Huang et al. 2022). MiR-330-5p/3p levels can be regulated by MALAT1 and it has already been shown that MALAT1 can sponge miR-330-5p (Shi et al. 2021). The functions of miR-330-5p and miR-330-3p in various types of cancer, not yet taking into account the interaction with MALAT1, have been discussed in a recent review article (Jafarzadeh et al. 2022).

**Table 10:** Expression levels of miRNAs targeting i.a. T*YMS* mRNA in cancer

miRNA	Level^†^	Cancers^‡^	References
miR-330-3p	Down	CRC tissues and HCT116, SW480 cell lines	Wang et al. (2022b)
	Down	CRC tissues: 5FU resistant vs sensitive (after surgery)	Gao et al. (2021a)
	Down	CRC tissues and SW480, SW620 cell lines	Huang et al. (2020b)
	Down	CRC tissues and HCT116, HT29, SW620, SW480 cell lines	Xu et al. (2017)
	Down	GC tissues and cell lines	Ma et al. (2020)
	Down	GC tissues and cell lines	Guan et al. (2016)
	Up	ESCC: two cell lines	Meng et al. (2015)
	Down	LSCC tissues and cell lines	Cheng et al. (2020a)
	Down	LSCC tissues and two cell lines	Fan and Zhu (2022)
	Down	OSCC tissues	Qian et al. (2021)
	Up	NSCLC tissues	Wang et al. (2021e)
	Up	NSCLC primary tissues with vs without brain metastasis	Wei et al. (2019b)
	Up	NSCLC tissues and four cell lines (i.a. A549)	Chen et al. (2019b)
	Up	NSCLC tissues	Liu et al. (2015)
	Up	BRCA tissues, four cell lines (i.a. MCF-7, MDA-MB-231)	Ji et al. (2021)
	Up	BRCA tissues (ER/HER2 ±), metastatic vs non-metastatic	Zhang et al. (2019b)
	Up	BRCA tissues (ER/PR ±)	Wang et al. (2018b)
	Down	BRCA (TNBC) tissues (stage I–III)	He et al. (2020)
	Up	HCC tissues and four cell lines	Zhao et al. (2019b)
	Up	HCC tissues, TNM stage II + III vs I, six cell lines	Hu et al. (2017a)
	Up	PDAC cell lines (i.a. PANC-1, SW 1990)	Xiong et al. (2019)
	Down	OVCA tissues, stage III–IV vs I–II, three cell lines	Cai et al. (2021)
	Down	PCa tissues and four cell lines (i.a. PC-3, DU145)	Li et al. (2020b)
	Down	PCa: four cell lines (i.a. PC-3, DU145)	Lee et al. (2009)

^†^,^‡^For description see footnote to Table 2

Using A549 cells, it was shown that miR-330-3p can also target *PTEN* mRNA and consequently promote AKT phosphorylation, which partly explains the selective pressure to increase miR-330-3p levels in NSCLC (Wang et al. 2021e). In turn, in highly invasive triple negative breast cancers, miR-330-3p targets the proto-oncogene c-Myc (He et al. 2020). Moreover, in prostate cancer cells, miR-330-3p was found to act as a tumor suppressor that targets the 3’UTR of mRNA encoding the transcription factor E2F1 (Lee et al. 2009). Thus, it can be seen that the selection pressure in cancer cells to decrease or increase the level of this miRNA is strongly dependent on the type of tumor.

Although miR-330-3p can target a variety of tumor suppressor genes, including *PTEN* and *PDCD4*, and is therefore up-regulated in various cancer types (see Table 11). In CRCs, however, miR-330-3p is down-regulated and targets *TYMS* mRNA and *HK2* mRNA (Gao et al. 2021a). *HK2* encodes Hexokinase 2 catalyzing the rate-limiting step of glucose metabolism and is therefore an enzyme that is highly expressed in rapidly growing cancer cells (Pedersen 2007; Sun et al. 2018b).

**Table 11:** Major genes targeted by miR-330-3p in various types of cancer

Cancers	Targeted gene products^†^	References
Cancers with down-regulation of miR-330-3p		
	*Acting as oncogenes or tumor promoters*	
CRC	TYMS, *HK2*	Xu et al. (2017); Gao et al. (2021a)
GC	*MSI1*, *PRRX1*	Guan et al. (2016); Ma et al. (2020)
LSCC	*SLC7A11*, *TRA2B*	Fan and Zhu (2022); Cheng et al. (2020a)
OSCC	*GLS*	Qian et al. (2021)
BRCA	*MYC*	He et al. (2020)
PCa	*BMI1*, *E2F1*	Li et al. (2020b); Lee et al. (2009)
	*Acting both as oncogenes and tumor suppressors, or unclassified)*	
CRC	*MYO6*, *PFN1*	Wang et al. (2022b); Huang et al. (2020b)
NSCLC	*GRIA3*	Wei et al. (2019b)
OVCA	*RIPK4*	Cai et al. (2021)
Cancers with up-regulation of miR-330-3p		
	*Acting as tumor suppressors*	
ESCC	*PDCD4*	Meng et al. (2015)
NSCLC	*EGR2*, *PTEN*, *RASSF1A*	Liu et al. (2015); Wang et al. (2021e); Chen et al. (2019b)
BRCA	*PDCD4*	Ji et al. (2021)
HCC	*BTG1*, *ING4*	Zhao et al. (2019b); Hu et al. (2017a)

^†^mRNAs encoding the following proteins: *BMI1* encodes BMI1 Proto-Oncogene, Polycomb Ring Finger; *BTG1* is a tumor suppressor gene that codes for BTG Anti-Proliferation Factor 1; *EGR2* is a tumor suppressor gene that codes for transcription factor Early Growth Response 2; *GLS* encodes GLS1 (Glutaminase) which promotes proliferation as opposed to proliferation-inhibiting GLS2 (Kim and Kim 2013); *GRIA3* encodes Glutamate Ionotropic Receptor AMPA Type Subunit 3; *HK2* encodes Hexokinase 2 catalyzing the rate-limiting step of glucose metabolism and is highly expressed in rapidly growing cancer cells; *ING4* encodes tumor suppressor protein that can bind p53; *MSI1* encodes Musashi RNA-binding protein that is involved in tumorigenesis; *MYC* encodes transcription factor MYC Proto-Oncogene (c-Myc); *MYO6* encodes motor protein Myosin VI; *PFN1* encodes Profilin 1 that binds actin monomers and mediates actin polymerization but also inhibit formation of IP3 from PIP2; *PDCD4* encodes programmed cell death protein 4 acting as a tumor suppressor; *PRRX1* codes for Paired Related Homeobox 1 protein that can promote the EMT process in cancer cells (Du et al. 2021); *RASSF1A* is a tumor suppressor gene encoding Ras Association Domain Family Member 1; *RIPK4* encodes Receptor Interacting Serine/Threonine Kinase 4 and acts as a tumor suppressor or promoter depending on the type of cancer (Xu et al. 2020c); *SLC7A11* encodes Cystine/Glutamate Transporter (Koppula et al. 2021); *TRA2B* is an oncogene encoding Transformer 2 β Homolog

### MiR-375-3p

Also for miR-375-3p there is a miRNA response element in the 3’UTR of *TYMS* mRNA (see Table 1) and overexpression of miR-375 mimics in CRC cell lines increased their sensitivity to cytotoxic activity of 5FU in vitro and in tumor-bearing mice (Xu et al. 2020b). As the transcription factor FOXM1 has been shown to up-regulate *TYMS* expression in CRCs (Varghese et al. 2019), it is wort noting that miR-375-3p also targets *FOXM1* mRNA (Chen et al. 2020b).

MiR-375-3p is down-regulated in CRC and analysis of clinical data showed a statistically significant reduction in miR-375-3p levels in stages III–IV compared to stages I–II (Mao et al. 2016), which is inverse to higher YAP1 levels in stages III–IV than in stages I–II (Sun et al. 2019b). Notes that miR-375-3p targets YAP1, a nuclear effector of the Hippo pathway, and the down-regulation of miR-375-3p in CRCs also leads to increased expression of Cyclin D1 and Survivin, which promotes proliferation and chemoresistance of cancer cells (Xu et al. 2019a). The works cited in Table 12 also contain data indicating a reduction in miR-375-3p levels in 5FU-resistant CRCs compared to 5FU-sensitive CRCs (Chen et al. 2020b; Xu et al. 2019a).

**Table 12:** Expression levels of miRNAs targeting i.a. T*YMS* mRNA in cancer

miRNA	Level^†^	Cancers^‡^	References
miR-375-3p	Down	CRC tissues, Caco2, HCT116, SW480, HT29 cell lines	Xu et al. (2020b)
	Down	CRC tissues	Liu et al. (2020a)
	Down	CRC tissues: 5FU-resistant vs sensitive	Chen et al. (2020b)
	Down	CRC tissues, Caco2, HCT116, SW480, HT29, SW620 lines	Xu et al. (2019a)
	Down	HCT8/FU (5FU resistant) vs HCT8 (parental sensitive)	Xu et al. (2019a)
	Down	HCT116/FU (5FU resistant) vs HCT116 (sensitive)	Xu et al. (2019a)
	Down	CRC tissues: 5FU-resistant vs sensitive	Xu et al. (2019a)
	Down	in serum of CRC patients	Huang et al. (2020a)
	Down	CRC tissues, HCT116, SW480, HT29, SW620 cell lines	Xu et al. (2016a)
	Down	CRC tissues, SW480, HT29, SW620, HCT116, HCT8	Mao et al. (2016)
	Down	CRC stage III–IV vs stage I–II	Mao et al. (2016)
	Down	CRC tissues	Wang et al. (2014b)
	Down	CRC tissues, HT29, SW620, HCT116 cell lines	Dai et al. (2012)
	Down	GC tissues and three cell lines	Liu et al. (2019b)
	Down	GC tissues and two cell lines	Huang et al. (2019b)
	Down	GC tissues and ten cell lines	Kang et al. (2018)
	Down	GC tissues	Chen et al. (2017d)
	Down	GC tissues	Yuan et al. (2018)
	Down	ESCC tissues and four cell lines	Li et al. (2021a)
	Down	ESCC tissues	Cheng et al. (2020b)
	Down	ESCC tissues and two cell lines	Xu et al. (2019b)
	Down	ESCC tissues and one cell line	Hu et al. (2017b)
	Down	ESCC tissues and cell lines	Kong et al. (2012)
	Down	LSCC and three cell lines	Chang et al. (2020)
	Up	LSCC: III/IV vs I/II TNM stage	Wu et al. (2016)
	Down	LSCC: III–IV vs I–II clinical stage	Guo et al. (2016)
	Down	LSCC tissues and two cell lines	Wang et al. (2016b)
	Down	LSCC tissues, UICC advanced III–IV vs early I–II stage	Luo et al. (2014)
	Down	OSCC tissues and four cell lines	Tong et al. (2021)
	Down	OSCC tissues and four cell lines	Wu et al. (2017)
	Down	OSCC tissues, with vs without lymph node metastasis	Zhang et al. (2017)
	Down	OSCC tissues	Shi et al. (2015)
	Down	NSCLC tissues and A549, H1299 cell lines	Jin et al. (2021)
	Down	NSCLC tissues, stages IV vs III vs II vs I	Chen et al. (2017f)
	Down	NSCLC (SqCLC) tissues, stage III vs II	Chen et al. (2017g)
	Up	BRCA tissues (luminal A/B, HER2^+^), three cell lines	Guan et al. (2021)
	Down	HCC tissues and four cell lines (i.a. Huh7)	Xu et al. (2021)
	Down	HCC tissues and five cell lines	Li et al. (2021b)
	Down	HCC tissues and four cell lines (i.a. Huh7)	Li et al. (2021c)
	Down	HCC tissues and five cell lines (i.a. Huh7)	Li et al. (2018b)
	Down	HCC tissues	He et al. (2012)
	Down	HCC tissues	Liu et al. (2010)
	Down	PDAC tissues and two cell lines (PANC-1, SW 1990)	Yonemori et al. (2017)
	Up	PDAC tissues and ten cell lines (i.a. PANC-1, SW 1990)	Yang et al. (2016)
	Down	PDAC tissues	Zhou et al. (2014b)
	Down	PDAC tissues	Song et al. (2013a)
	Down	PDAC tissues and four cell lines (i.a. PANC-1, SW 1990)	Song et al. (2013b)
	Down	CeCa tissues (stage I-IV) and four cell lines	Cao et al. (2021)
	Up	CeCa: PTX-resistant vs pre-chemotherapy tissues	Shen et al. (2013)
	Down	CeCa tissues, FIGO stage IIA vs IB1/IB2	Wang et al. (2011)
	Down	OVCA tissues and four cell lines	Shu et al. (2021)
	Up	PCa tissues	Porzycki et al. (2018)

^†^,^‡^For description see footnote to Table 2

In contrast, in breast cancers (both luminal A/B and HER2-positive) the up-regulated mir-375-3p targets the mRNA encoding the tumor suppressor FOXO1 (forkhead box protein O1), which activates the p53 signaling pathway and indeed p53 tumor suppressor was found to be decreased along with FOXO1 in breast cancer cell lines (Guan et al. 2021). Also in paclitaxel-resistant cervical cancer, miR-375-3p targeting *CDH1* mRNA encoding E-cadherin was found to be up-regulated, therefore miR-375-3p may facilitate the EMT process of cervical cancer cells (Shen et al. 2013, 2014).

The direct interaction of miR-375-3p with the miRNA response element in MALAT1 was supported by the results of luciferase reporter assays performed with 293 T fibroblast cells, while the results of the RNA pull-down assays using hepatocellular carcinoma cells showed a direct interaction of MALAT1-miRNA dependent on the miR-375-3p seed sequence (Zhao et al. 2020a).

**Fig. 3:**
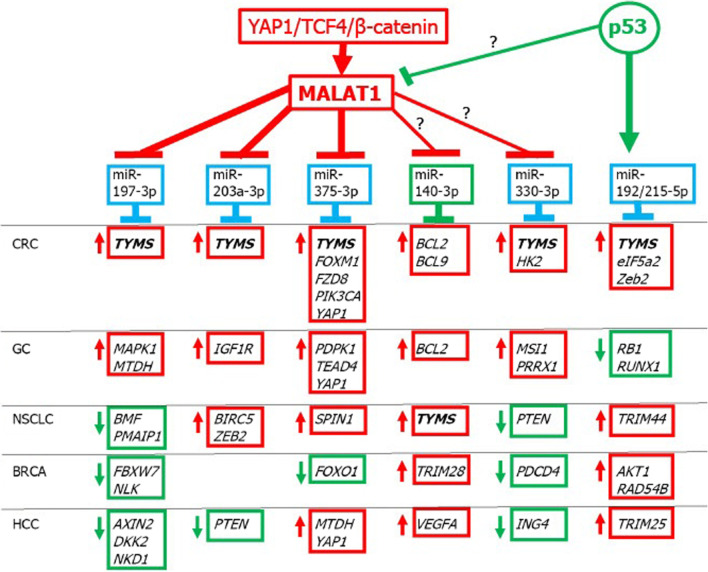
MALAT1-miRNAs network regulating the expression level of *TYMS* along with other cancer-related genes in various types of cancer. Cancer type designation: CRC, colorectal cancer; GC, gastric cancer; NSCLC, non-small cell lung cancer; BRCA, breast cancer; HCC, hepatocellular carcinoma. Red indicates oncogenic effects, green indicates tumor suppressor effects, blue indicates tumor suppressor or oncogenic effects depending on the type of cancer. YAP1 in complex with TCF4/β-catenin upregulates the expression of the *MALAT1* gene in CRC and HCC (Sun et al. 2019b; Wang et al. 2014a)

As a brief summary, Tables 12 and 13 show that down-regulation of miR-375-3p targeting *FOXM1*, *TYMS*, *YAP1*, *PIK3CA*, *FZD8* can promote 5FU resistance of CRC cells, tumor growth and CRC metastasis by activating the PI3K/AKT and WNT/β-catenin signaling pathways. In CRC cells, YAP1 promotes proliferation and inhibits apoptosis by upregulating Survivin (Xu et al. 2019a). Besides CRCs, miR-375-3p targets the Hippo pathway effector YAP1 also in other cancers such as GC, HCC and OVCA (see Table 13 and Fig. 3).

**Table 13:** Major genes targeted by miR-375-3p in various types of cancer

Cancers	Targeted gene products^†^	References
Cancers with down-regulation of miR-375-3p		
	*Acting as oncogenes or tumor promoters*	
CRC	TYMS, *CBX3*, *FOXM1*,	Xu et al. (2020b); Liu et al. (2020a); Chen et al. (2020b);
	*FZD8*, *PIK3CA*, YAP1	Xu et al. (2016a); Wang et al. (2014b Xu et al. (2019a
ESCC	*MTDH*	Hu et al. (2017b)
GC	*PDPK1*, *TEAD4*, YAP1,	Chen et al. (2017d); Kang et al. (2018)
	*YBX1*, *YWHAZ*	Huang et al. (2019b); Liu et al. (2019b)
OSCC	*SLC7A11*	Wu et al. (2017)
NSCLC	*SPIN1*	Jin et al. (2021)
HCC	*ERBB2*, *HMGA2*, *JAK2*	Li et al. (2018b); Xu et al. (2021); Li et al. (2021b)
HCC	*MTDH*, *PDGFC*, YAP1	He et al. (2012); Li et al. (2021c); Liu et al. (2010)
HCC CSCs	YAP1	Zhao et al. (2020a)
PDAC	*PDPK1, ZFP36L2*	Zhou et al. (2014b); Song et al. (2013a); Yonemori et al. (2017)
OVCA	YAP1	Shu et al. (2021)
	*Acting both as oncogenes and tumor suppressors, or unclassified*	
CRC	*KLF4*, *SP1*	Mao et al. (2016); Xu et al. (2019a)
GC	*CCN2*	Kang et al. (2018)
ESCC	*HMGB1*, *IGF1R*, *SESN3*,	Cheng et al. (2020b); Kong et al. (2012); Li et al. (2021a)
	*SP1*	Xu et al. (2019b)
LSCC	*HNF1B*, *IGF1R*	Chang et al. (2020); Luo et al. (2014)
OSCC	*KLF5*, *IGF1R*, PAX6	Shi et al. (2015); Zhang et al. (2017); Tong et al. (2021)
CeCa	*SP1*	Wang et al. (2011)
Cancers with up-regulation of miR-375-3p		
	*Acting as tumor suppressors*	
BRCA	*FOXO1*	Guan et al. (2021)
CeCa	*CDH1*	Shen et al. (2014)
	*Acting both as oncogenes and tumor suppressors, or unclassified*	
PDAC	*HOXB3*	Yang et al. (2016)

^†^mRNAs encoding the following proteins: *CBX3* codes for protein involved in transcriptional silencing (upregulated in cancers); *CCN2* codes for connective tissue growth factor; *CDH1* is a tumor suppressor gene encoding E-cadherin; *ERBB2* codes for HER-2 Receptor Tyrosine Kinase; *FOXO1* is a tumor suppressor gene; *FZD8* encodes Frizzled Class Receptor 8 which is a receptor for Wnt proteins (Sun et al. (2021); *HMGA2* encodes HMG AT-Hook Protein 2, which functions in the regulation of the cell cycle; *HMGB1* encodes HMG Box 1, which has both oncogenic and tumor suppression functions; *HNF1B* encodes Hepatocyte Nuclear Factor 1-β and functions as a tumor suppressor gene or oncogene (Chandra et al. (2021); *HOXB3* encodes transcription factor Homeobox B3 (Li et al. (2019b); *JAK2* encodes Janus Kinase 2 phosphorylating a tyrosine residue; *KLF4* is a tumor suppressor gene encoding Kruppel Like Factor 4, but may also act as an oncogene depending on the cellular context; *KLF5* encodes Kruppel Like Factor 5 and acts as an oncogene or tumor suppressor gene depending on the cellular context; *PAX6* is a tumor suppressor gene encoding transcription factor Paired Box 6; *PDGFC* codes for Platelet Derived Growth Factor C (promotes angiogenesis); *PDPK1* encodes 3-Phosphoinositide-Dependent Protein Kinase-1 (Domrachev et al. (2021); *PIK3CA* is an oncogene encoding PI3K, subunit p110α; *SESN3* encodes sestrin3, stress-induced protein; *SP1* encodes Sp1 Transcription Factor that regulates oncogenes and tumor suppressor genes; *SPIN1* encodes Spindlin 1 and is considered an oncogene (Janecki et al. (2018); *TEAD4* is an oncogene encoding TEA Domain Transcription Factor 4 (Hippo pathway); *YBX1* is an oncogene encoding Y-Box Binding Protein 1; *YAP1* is an oncogene encoding Yes1 Associated Transcriptional Regulator (Hippo pathway); *ZFP36L2* is an oncogene encoding ZFP36 Ring Finger Protein Like 2; *YWHAZ* is functioning as an oncogene and codes for an adapter protein belonging to the 14-3-3 family

### MiR-433-3p

In turn, overexpression of miR-433-3p in human cervical cancer HeLa cells resulted in a reduction of *TYMS* mRNA and protein levels, and sensitized cells to treatment with 5FU (Gotanda et al. 2013). The set of oncogenes targeted by miR-433-3p that is down-regulated in various types of cancer (Table 14) is listed in Table 15. From this overview, it can be seen that miR-433-3p targeting mRNA encoding the cAMP Response Element-Binding protein 1 (CREB1) has been found in cancer cells of various types including CRC, bladder cancer and HCC (Yan et al. 2018; Xu et al. 2016b; Yang et al. 2013).

**Table 14:** Expression levels of miRNAs targeting i.a. T*YMS* mRNA in cancer

miRNA	Level^†^	Cancers^‡^	References
miR-433-3p	Down	CRC tissues, LoVo, SW620, SW480, HCT116 cell lines	Zhang et al. (2018)
	Down	CRC: stage II vs stage I	Zhang et al. (2018)
	Down	CRC tissues, SW480, LoVo, HT29, Caco-2, SW620 lines	Li et al. (2018c)
	Down	CRC tissues and cell lines	Yan et al. (2018)
	Down	CRC tissues, SW620, HCT116, SW480, LoVo, HT29 lines	Li et al. (2017c)
	Down	GC: recurrent vs non-recurrent	Wang et al. (2021c)
	Down	GC tissues: stage IV vs stage II	Guo et al. (2013)
	Down	ESCC tissues and two cell lines	Li and Li (2021)
	Down	ESCC tissues	Cheng et al. (2020b)
	Down	OSCC tissues	Liu et al. (2020b)
	Down	OSCC tissues, TNM stage III/IV vs I/II	Wang et al. (2017)
	Down	OSCC tissues and five cell lines	Wang et al. (2015)
	Down	NSCLC tissues and A549, H1975 cell lines	Yu et al. (2022)
	Down	NSCLC tissues and A549, H522 cell lines	Guo et al. (2022)
	Down	NSCLC tissues and A549, H1299, H23, H1581 cell lines	Zhang et al. (2021)
	Down	NSCLC tissues	Liu et al. (2020c)
	Down	NSCLC tissues	Li et al. (2019a)
	Down	NSCLC tissues and four cell lines (A549, H460, H522)	Liu et al. (2018)
	Down	BLCA tissues and five cell lines (i.a. T24)	Wang et al. (2020b)
	Down	BLCA tissues and three cell lines (i.a. T24)	Xu et al. (2016b)
	Down	HCC tissues, stage III/IV vs I/II, four cell lines	Song et al. (2021)
	Down	HCC tissues	Ma et al. (2021a)
	Down	PDAC tissues and two cell lines (PANC-1, SW 1990)	Zhou et al. (2021b)
	Down	CeCa tissues and four cell lines	Liang et al. (2017)
	Down	OVCA tissue	Liang et al. (2016)

^†^,^‡^For description see footnote to Table 2

**Table 15:** Major genes targeted by miR-433-3p in various types of cancer

Cancers	Targeted gene products^†^	References
Cancers with down-regulation of miR-433-3p		
	*Acting as oncogenes or tumor promoters*	
CRC	*CREB1*, *HOXA1*, *MACC1*, *MAPK8*	Yan et al. (2018); Li et al. (2018c); Li et al. (2017c)
GC	*KRAS*	Guo et al. (2013)
ESCC	*REV3L*	Li and Li (2021)
OSCC	*HDAC6*, *PAK4*, *PTK2*	Wang et al. (2015); Liu et al. (2020b); Wang et al. (2017)
NSCLC	*E2F3*, *LPCAT1*, *SMAD2*, *NUCKS1*	Liu et al. (2018); Guo et al. (2022); Li et al. (2019a); Yu et al. (2022)
BLCA	*CREB1*	Xu et al. (2016b)
HCC	*CREB1*	Yang et al. (2013)
PDAC	*GOT1*	Zhou et al. (2021b)
CeCa	TYMS, *MTDH*	Gotanda et al. (2013); Liang et al. (2017)
	*Acting both as oncogenes and tumor suppressors, or unclassified*	
CRC	*CCAR1*	Yan et al. (2018)
GC	*ATG5*, *ATG12*	Wang et al. (2021c)
ESCC	*HMGB1*	Cheng et al. (2020b)
NSCLC	*TIPRL*	Zhang et al. (2021)
BLCA	*CCAR1*	Wang et al. (2020b)
HCC	*CBX3*, *FXYD3*	Song et al. (2021); Ma et al. (2021a)
OVCA	*NOTCH1*	Liang et al. (2016)

^†^mRNAs encoding the following proteins: *ATG5* and *ATG12* code for Autophagy-Related Proteins 5 and 12, respectively; *CBX3* encodes Chromobox Protein Homolog 3 and functions as both an oncogene and a tumor suppressor; *CCAR1* codes for Cell Division Cycle And Apoptosis Regulator 1 that functions as a transcriptional coactivator; *CREB1* codes for transcription factor cAMP Response Element Binding Protein 1; *E2F3* is an oncogene coding for E2F Transcription Factor 3; *FXYD3* codes for Sodium/Potassium-Transporting ATPase Subunit FXYD3; *GOT1* codes for Glutamic-Oxaloacetic Transaminase 1 that functions as a tumor promoter in pancreatic cancer growth; *HDAC6* encodes Histone Deacetylase 6; *HOXA1* is an oncogene coding for transcription factor Homeobox A1 (Li et al. 2019b); *KRAS* encodes KRAS Proto-Oncogene (GTPase K-Ras); *LPCAT1* is an oncogene encoding Lysophosphatidylcholine Acetyltransferase 1 (Wei et al. 2019c); *MACC1* codes for Metastasis-Associated in Colon Cancer protein 1; *MAPK8* codes for Mitogen-Activated Protein Kinase 8 and is involved in colon cancer chemoresistance (Wu et al. 2019); *NOTCH1* encoding Notch Receptor 1 acts as an oncogene or tumor suppressor gene, depending on the cellular context; *NUCKS1* is recognized as oncogene encoding Nuclear Casein Kinase and cyclin dependent Kinase Substrate 1 that is involved in DNA repair and promotes proliferation, invasion and migration of NSCLC (Zhao et al. 2020c); *PAK4* gene codes for P21 (RAC1) Activated Kinase 4, serine/threonine protein kinase; *PTK2* encodes non-receptor Protein Tyrosine Kinase 2, also known as Focal Adhesion Kinase; *REV3L* codes for catalytic subunit of the DNA polymerase zeta complex, involved in the chemoresistance of ESCC (Zhu et al. 2016); *SMAD2* encodes the SMAD Family Member 2 protein, which can act as an oncogenic protein in the TGF-β pathway (Pefani et al. 2016); *TIPRL* codes for TOR Signaling Pathway Regulator that was found as metastasis suppressor in gastric cancer

### MiR-1307-3p

Mir-1307-3p targets a variety of tumor suppressor genes and is rather up-regulated in various types of cancer (see Tables 16, 17). However, miR-1307-3p also directly targets 3’UTR of *TYMS* mRNA and is significantly involved in the regulation of TS protein levels, as in CRC patients with low miR-1307-3p levels (due to T > C SNP in the terminal-loop of pre-miRNA) (Tang et al. 2015) elevated TS levels were reported along with insensitivity to capecitabine chemotherapy (Chen et al. 2017a). Moreover, relatively high levels of miR-1307-3p and low levels of *TYMS*-encoded protein were found in Caco-2 and HCT116 cell lines, while low levels of miR-1307-3p along with high levels of *TYMS*-encoded protein were found in SW480, LoVo and DLD1 cell lines (Chen et al. 2017a). This suggests a significant involvement of miR-1307-3p in the regulation of *TYMS* expression in at least some colon cancers. However, this effect does not appear to be of any importance in other cancers. For example, in the early stages of breast cancer of a various molecular subtypes (luminal, triple negative), miR-1307-3p significantly targets the mRNA encoding the SMYD4 protein (Han et al. 2019) identified as a tumor suppressor in breast cancer (Hu et al. 2009).

**Table 16:** Expression levels of miRNAs targeting i.a. T*YMS* mRNA in cancer

miRNA	Level^†^	Cancers^‡^	References
miR-1307-3p	Down	CRC tissues, HT29, SW480, SW620, HCT116 cell lines	Zheng et al. (2019a)
	Down	CRC cell lines: LoVo, DLD1, SW620, SW480	Chen et al. (2017a)
	Up	SW480 and SW620 colon cancer cell lines	Yue et al. (2020)
	Up	GC tissues and cell lines	Ma et al. (2021b)
	Up	BLCA tissues	Wang et al. (2021b)
	Up	BRCA tissues (stage I), cell lines (MCF-7, MDA-MB-231)	Han et al. (2019)
	Up	HCC tissues	Wang et al. (2019b)
	Up	HCC tissues and four cell lines	Chen et al. (2019a)
	Up	OVCA: PTX-resistant vs sensitive cancer tissue	Zhou et al. (2015)
	Up	PCa tissue and five cell lines (i.a. PC-3, DU145)	Qiu and Dou (2017)

^†^,^‡^For description see footnote to Table 2

**Table 17:** Major genes targeted by miR-1307-3p in various types of cancer

Cancers	Targeted gene products†	References
Cancers with down-regulation of miR-1307-3p		
	*Acting as oncogenes or tumor promoters*	
CRC	TYMS	Chen et al. (2017a)
	*Acting as tumor suppressors*	
CRC	*ISM1*	Zheng et al. (2019a); Yue et al. (2020)
Cancers with up-regulation of miR-1307-3p		
	*Acting as tumor suppressors*	
CRC	*TRARG1*	Zheng et al. (2019a)
GC	*DAB2IP*	Ma et al. (2021b)
BLCA	*SMG1*	Wang et al. (2021b)
BRCA	*SMYD4*	Han et al. (2019)
HCC	*DAB2IP*, *ISM1*	Chen et al. (2019a); Wang et al. (2019b)
OVCA	*DAPK3*	Zhou et al. (2015)
PCa	*FOXO3*	Qiu and Dou (2017)

^†^mRNAs encoding the following proteins: *DAB2IP* is a tumor suppressor gene encoding DAB2 Interacting Protein, a Ras GTPase-activating protein; *DAPK3* is a tumor suppressor gene that codes for serine/threonine Death Associated Protein Kinase 3; *FOXO3* is a tumor suppressor gene encoding a transcription factor that activates genes encoding i.a. pro-apoptotic BIM and PUMA; *ISM1* codes for Isthmin 1 that acts as an angiogenesis inhibitor; *SMG1* is a tumor suppressor gene encoding a protein that can enhance p53 activity (Gewandter et al. 2011); *SMYD4* is a tumor suppressor gene involved in the development of breast cancer; *TRARG1* codes for Trafficking Regulator of GLUT4

In summary, a series of at least a few miRNAs (see Table 1 and Fig. 2) acting directly on *TYMS* mRNA fine-tune TS enzyme protein levels according to cell needs and may contribute to cancer cell resistance to the cytotoxic effects of 5FU. In addition, at least two lncRNAs, MALAT1 and TUG1, affect TS levels by sponging the respective miRNAs (see Table 18). Other lncRNAs, such as HOTAIR and XIST, also appear to affect TS levels and the sensitivity of cancer cells to 5FU, although the mechanisms of action are still poorly understood.

**Table 18:** lncRNA MALAT1 sponging miRNAs targeting *TYMS*

lncRNA	Confirmed or putative binding sites for miRNA†	References
MALAT1	*Confirmed as ceRNA for miRNAs:*	
	miR-197-3p at 2775–2781, 2814–2820, 2854–2864	Yang et al. (2019)
	miR-203a-3p at 5414–5419, 6400–6405	Yu et al. (2020); Zhang et al. (2019a); Chen et al. (2017b)
	miR-375-3p at 772–778	Zhao et al. (2020a)
MALAT1	*Presumably can sponge miRNAs:*	
	miR-140-3p at 683–689, 4525–4531, 8737–8743	–
	miR-330-3p at 8439–8445, and other	–
TUG1	*Confirmed as ceRNA for miRNAs:*	
	miR-140-3p at 2096–2101, 6438–6443, 6728–6733	Yuan et al. (2021)
	miR-197-3p at 1438–1444, 2098–2103, 3549–3554	Wang et al. (2019a); Tang et al. (2018)

^†^Sites in MALAT1 and TUG1 (transcript variant 8) according to the GenBank sequence accession numbers NR_002819.4, NM_001398480.1, respectively; sites confirmed by the luciferase reporter assay are underlined

## MiRNAs targeting *DPYD* mRNA

Two highly homologous miRNAs, miR-27a-3p and miR-27b-3p, have been shown to directly reduce the levels of *DPYD* mRNA and DPD protein in CRC cells, and ectopic expression of miR-27a-3p and miR-27b-3p significantly increased the sensitivity of cancer cells to the cytotoxic effect of 5FU (Offer et al. 2014). Another report showed that ectopic overexpression of miR-302b-3p led to an increase in the sensitivity of HCC cell lines to 5FU by negatively regulating DPD protein levels, as well as inhibiting entry into the S phase of the cell cycle and promoting apoptosis by targeting mRNA encoding the anti-apoptotic protein MCL-1 (Cai et al. 2015). MiR-494-3p was also found to directly target *DPYD* mRNA and miR-494-3p was shown to be reduced in CRC cells selected for 5FU resistance, compared to 5FU sensitive parental cells, while miR-494 mimic caused restoration of cell chemosensitivity to 5FU (Chai et al. 2015).

It should also be noted that the levels of enzymes encoded by the *TYMS*, *TYMP* and *DPYD* genes are indirectly regulated by miR-21-5p targeting the human mutS homolog2 (hMSH2) in CRC cells (Deng et al. 2014a). Finally, it is worth mentioning that both miR-215-5p and miR-21-5p, regulating the *TYMS*-encoded protein directly or indirectly, are included in a set of six miRNAs whose levels were tested in CRC surgical specimens for verification as a diagnostic tool to predict which stage II CRC patients may benefit from chemotherapy following radical surgery for CRC. That clinical trial project was registered at ClinicalTrial.gov as NCT02635087. According to published data, miR-215-5p along with miR-103a-3p and miR-143-5p, both alone and in combination, have proven to be reliable stage II CRC biomarkers that can help stratify patients into risk groups and identify patients who could potentially benefit from postoperative chemotherapy (Caritg et al. 2016).

## Conclusions and future perspectives

Putting it all together, it can be seen that, at least in colon cancer, several miRNAs targeting *TYMS* mRNA have been identified, the levels of which can be regulated to varying degrees by long non-coding RNAs, creating a complex regulatory network. Due to their negative regulation by MALAT1, which can consequently increase the level of the TS enzyme and decrease the susceptibility to 5FU treatment, these miRNAs can be divided into three groups (Fig. 3).

The first group of miRNAs targeting *TYMS* mRNA consists of miR-197-3p, miR-203a-3p and miR-375-3p which are negatively regulated by MALAT1 as confirmed experimentally. The levels of these miRNAs are actually reduced in colon and gastric cancers. On the other hand, the selection pressure to increase miR-197-3p levels may be suggested in non-small cell lung cancer, bladder cancer, breast cancer and hepatocellular carcinoma (Table 6 and Fig. 2), presumably because miR-197-3p also targets mRNAs encoding proteins that function as tumor suppressors in these types of cancer, such as apoptosis initiators in lung cancers (Fiori et al. 2014) or negative regulators of the Wnt/β-catenin pathway in hepatocellular carcinoma (Hu et al. 2018a). On the other hand, decreased levels of miR-375-3p were found in most of the cancer types shown in Fig. 2, and apart from the targeted *TYMS* mRNA, the targeting of *YAP1* mRNA by miR-375-3p has also been found in various cancers (Table 13).

The second group of miRNAs targeting *TYMS* mRNAs consists of miR-140-3p and miR-330-3p, which could potentially be sponged by MALAT1, but the inhibition of these miRNAs by MALAT1 has not yet been supported by experimental results. While miR-140-3p levels have been found to be down-regulated in all the cancer types shown in Fig. 2, miR-330-3p levels have also been found to be reduced in colon and gastric cancers, but the influence of selection pressure on the elevation of miR-330-3p levels may be suggested in non-small cell lung cancer, breast cancer and hepatocellular carcinoma, in which miR-330-3p seems to be significantly involved also in targeting tumor suppressor genes such as *PTEN*, *PDCD4*, and *ING4* (Table 11 and Fig. 3).

The third group of miRNAs targeting TYMS mRNAs consists of miR-192-5p, miR-215-5p, miR-433-3p and miR-1307-3p whose seed sequences do not recognize complementary miRNA response elements within MALAT1. MiR-192-5p and miR-215-5p are up-regulated by p53 in response to DNA damage and target mRNAs encoding i.a. proliferation promoting proteins including *TYMS* mRNA (Fig. 3). MiR-192/215-5p also target mRNA encoding the transcriptional corepressor ZEB2 (Chen et al. 2017c), which is involved in the transformation of epithelial cancer cells into cells capable of migration, invasion and metastasis. MiR-192/215-5p are down-regulated in most types of cancers (Fig. 2), but in the invasive and metastatic stages of gastric cancers there appears to be a selection pressure on the up-regulation of these miRNA, presumably because they also target mRNA encoding a significant tumor suppressor and Retinoblastoma-associated protein (Chen et al. 2017e). In turn, the potential involvement of miR-433-3p and miR-1307-3p in the regulation of TS levels in colon cancers does not seem to be well documented. Although miR-433 is down-regulated in all the cancer types analyzed (Fig. 2), miR-433-3p targeting *TYMS* mRNA and its contribution to increasing 5FU susceptibility was demonstrated primarily in cervical cancer HeLa cells (Gotanda et al. 2013). To conclude this part of the discussion, although one report showed that miR-1307-3p targets *TYMS* mRNA in colon cancer cells (Chen et al. 2017a), the biological significance of this interaction is uncertain as miR-1307-3p is elevated in most of the cancer types analyzed here (Fig. 2).

**Fig. 4:**
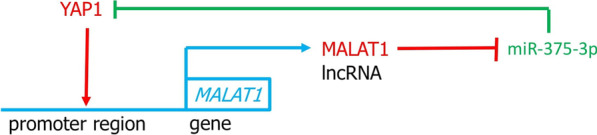
Hypothetical feedback between lncRNA MALAT1 and YAP1 via miR-375-3p enhancing *MALAT1* gene expression

Considering the putative MALAT1-miRNAs interaction network presented in Fig. 3, a potential positive feedback loop is seen to the upregulate MALAT1 expression in colon cancer as well as in hepatocellular carcinoma (Fig. 4). In both colon cancer and hepatocellular carcinoma, it was found that YAP1 can complex with TCF4/β-catenin at the promoter region of the *MALAT1* gene and enhance the expression of this gene (Sun et al. 2019b; Wang et al. 2014a). Further, the lncRNA MALAT1 can directly interact with miR-375-3p as revealed by the results of the RNA pull-down assay in Huh7 hepatocellular carcinoma cells, thus counteracting the down-regulation of YAP1 by miR-375-3p (Zhao et al. 2020a). Thus, YAP1, by activating the *BIRC5* gene encoding the anti-apoptotic Survivin, contributes, along with the increase in the TS enzyme level, to reducing the sensitivity of colorectal cancer cells to the 5FU treatment (Xu et al. 2019a). YAP1 also promotes the EMT process which enables the cancer metastasis process (Ling et al. 2017). Interestingly, the direct interaction of YAP1 with the TCF4/β-catenin complex was also found in the nuclei of placenta-derived mesenchymal stem cells (Feng et al. 2020). It would be valuable to try to determine whether the involvement of YAP1 in the upregulation of *MALAT1* gene expression is unique in colon cancer and hepatocellular carcinoma, or whether a similar upregulation mechanism of *MALAT1* gene expression is involved in various types of cancer. The exact mechanism leading to aberrant expression of MALAT1 is still unknown, but various transcription factors, coactivators and RNA-binding proteins may be involved (Xu et al. 2022). Therefore, it may also be the starting point for the search for small molecule inhibitors of the YAP1/TCF4/β-catenin transcription complex (Yong et al. 2021; Yan et al. 2020a; Tang et al. 2019b). This could be a fruitful area for further research in the context of the lack of approved therapy targeting the abnormal overexpression of MALAT1 (Uthman et al. 2021). Presumably, at least partially suppressing MALAT1 overexpression by influencing the miRNA interaction network could both reduce tumor metastatic potential and increase the sensitivity of cancer cells to 5FU.

Finally, it would also be important to estimate the weight of the individual elements of the MALAT1-miRNAs network, taking into account their actual concentrations in a 5FU-sensitive cancer cell compared to a 5FU resistant cell, as this could contribute to the construction of a diagnostic tools to monitor the therapeutic process.

This article focuses on those non-coding RNAs that, by regulating the level of thymidylate synthase, can influence the sensitivity of cancer cells to 5FU treatment. It is worth noting, however, that the MALAT1 and miRNAs network outlined here regulating the level of the protein encoded by *TYMS* is rather a subset of a much larger set of non-coding RNA networks involving MALAT1 (Su et al. 2021; Poursheikhani et al. 2020). The effectiveness of 5FU therapy may also depend on other non-coding RNAs, not mentioned in this article, that may affect the concentration of fluoropyrymidines inside the cell, as well as affect glucose metabolism in the cancer cell (Marjaneh et al. 2019), DNA damage repair, cell cycle regulation, inducing and executing apoptosis. While the main goal of looking for interactions between non-coding RNAs in cancer cells is to open up new possibilities for diagnosis and therapy, identifying potential MALAT1 and miRNAs interaction networks is also interesting in itself, as like the Rosetta Stone mentioned earlier (Salmena et al. 2011), it points to complex mechanisms of fine-tuning the expression level of key genes involved in the proliferation of cancer cells.

## Data Availability

Not applicable.

## Abbreviations

5FU: 5-Fluorouracil
BLCA: Bladder cancer
BRCA: Breast cancer
CeCa: Cervical cancer
ceRNA: Competing endogenous RNA
CRC: Colorectal cancer
CSC: Cancer stem-like cell
DPD: Dihydropyrimidine dehydrogenase
EMT: Epithelial-mesenchymal transition
ESCC: Esophageal squamous cell carcinoma
GC: Gastric cancer
HCC: Hepatocellular carcinoma
lncRNA: Long non-coding RNA
LSCC: Laryngeal squamous cell carcinoma
LUAD: Lung adenocarcinoma
miRNA: MicroRNA
mRNA: Messenger RNA
NSCLC: Non-small-cell lung cancer
OSCC: Oral squamous cell carcinoma
OVCA: Ovarian cancer
PCa: Prostate cancer
PDAC: Pancreatic ductal adenocarcinoma
RISC: RNA-induced silencing complex
SqCLC: Squamous cell lung carcinoma
TNBC: Triple-negative breast cancer
TNM: Tumor-node-metastasis
TS: Thymidylate synthase
